# Tunneling current modulation in atomically precise graphene nanoribbon heterojunctions

**DOI:** 10.1038/s41467-021-22774-0

**Published:** 2021-05-05

**Authors:** Boris V. Senkovskiy, Alexey V. Nenashev, Seyed K. Alavi, Yannic Falke, Martin Hell, Pantelis Bampoulis, Dmitry V. Rybkovskiy, Dmitry Yu. Usachov, Alexander V. Fedorov, Alexander I. Chernov, Florian Gebhard, Klaus Meerholz, Dirk Hertel, Masashi Arita, Taichi Okuda, Koji Miyamoto, Kenya Shimada, Felix R. Fischer, Thomas Michely, Sergei D. Baranovskii, Klas Lindfors, Thomas Szkopek, Alexander Grüneis

**Affiliations:** 1 II. Physikalisches Institut, Universität zu Köln, Köln, Germany; 2 Rzhanov Institute of Semiconductor Physics, Novosibirsk, Russia; 3 Department of Physics, Novosibirsk State University, Novosibirsk, Russia; 4 Department für Chemie, Universität zu Köln, Köln, Germany; 5 Institut für Angewandte Physik der Universität Bonn, Bonn, Germany; 6 Skolkovo Institute of Science and Technology, Moscow, Russia; 7 Saint Petersburg State University, Saint Petersburg, Russia; 8 IFW Dresden, Dresden, Germany; 9 Helmholtz-Zentrum Berlin für Materialien und Energie (HZB), Berlin, Germany; 10 Center for Photonics and 2D Materials, Moscow Institute of Physics and Technology (MIPT), Dolgoprudny, Russia; 11 Russian Quantum Center, Moscow, Russia; 12 Faculty of Physics and Material Sciences Center, Philipps-Universität, Marburg, Germany; 13 Hiroshima Synchrotron Radiation Center, Hiroshima University, Higashi-Hiroshima, Japan; 14 Department of Chemistry, University of California at Berkeley, Berkeley, CA USA; 15 Materials Sciences Division, Lawrence Berkeley National Laboratory, Berkeley, CA USA; 16 Kavli Energy NanoSciences Institute at the University of California Berkeley and the Lawrence Berkeley National Laboratory, Berkeley, CA USA; 17 Department of Electrical and Computer Engineering, McGill University, Montreal, QC Canada

**Keywords:** Electronic devices, Sensors, Molecular self-assembly, Electronic properties and materials, Surfaces, interfaces and thin films

## Abstract

Lateral heterojunctions of atomically precise graphene nanoribbons (GNRs) hold promise for applications in nanotechnology, yet their charge transport and most of the spectroscopic properties have not been investigated. Here, we synthesize a monolayer of multiple aligned heterojunctions consisting of quasi-metallic and wide-bandgap GNRs, and report characterization by scanning tunneling microscopy, angle-resolved photoemission, Raman spectroscopy, and charge transport. Comprehensive transport measurements as a function of bias and gate voltages, channel length, and temperature reveal that charge transport is dictated by tunneling through the potential barriers formed by wide-bandgap GNR segments. The current-voltage characteristics are in agreement with calculations of tunneling conductance through asymmetric barriers. We fabricate a GNR heterojunctions based sensor and demonstrate greatly improved sensitivity to adsorbates compared to graphene based sensors. This is achieved via modulation of the GNR heterojunction tunneling barriers by adsorbates.

## Abstract (web summary)

Here, the authors characterize the spectroscopic and transport properties of heterojunctions composed of quasi-metallic and semiconducting graphene nanoribbons (GNRs) with different widths, showing a predominant quantum tunnelling mechanism. The GNR heterojunctions can also be used to realize adsorbate sensors with high sensitivity.

## Introduction

The significance of heterojunctions is established in semiconductor physics. Vertically grown compound semiconductor heterostructures such as GaAs/Al_*x*_Ga_1−*x*_As^1,2^ have been developed to enable high electron mobility transistors, light-emitting diodes, laser diodes, and solar cells. Tunneling through multiple potential barriers in superlattice heterostructures^3,4^ has found use in quantum cascade lasers^5^. More recently, van der Waals (vdW) heterostructures that are fabricated from monolayers of layered materials have attracted research interest^6,7^. Graphene nanoribbon (GNR) heterostructures differ from compound semiconductor and vdW heterostructures in that the bottom–up synthesis allows for the formation of one-dimensional (1D) lateral interfaces with atomic precision. This synthesis approach enables electronic band structure engineering, i.e., control of parameters such as the energy bandgap, the band offset, and the effective mass of carriers that are important for charge transport^8–15^. Conventional top–down fabrication methods such as lithography do not have the precision required for reproducible, atomically precise GNR heterojunctions that have well-defined, sharp band offsets. Thus, bottom–up GNR heterojunctions are promising for novel device concepts such as energy-efficient tunnel field-effect transistors (TFETs)^15–17^. TFETs based on GNR heterostructures might also be useful for chemical sensing devices. Yet progress is hampered by the absence of experiments that probe the electronic properties of GNR heterojunctions. There are two main obstacles for the fabrication of GNR heterojunction-based tunneling devices that are overcome in the present work. The first one is the random orientation of bottom–up synthesized GNRs that yield disconnected heterojunctions scattered over the substrate surface^12,14^. The second obstacle is the presence of significant Schottky barriers at the metal–GNR interface, which can determine the device performance^18,19^.

In the present work, we use the lateral fusion approach to fabricate a monolayer film consisting of aligned lateral heterojunctions of wide-bandgap armchair GNRs of *N* = 7 carbon atom width (7-AGNRs) and their quasi-metallic derivatives (mostly 14-AGNRs) on a stepped Au(788) surface. The GNR heterojunctions are comprehensively characterized by scanning tunneling microscopy (STM), angle-resolved photoemission spectroscopy (ARPES), and Raman spectroscopy and are integrated into a back-gated FET structure. This allows to perform ensemble studies of charge transport in GNR heterojunctions. We demonstrate that the current through the device has the characteristic dependencies on bias voltage, charge carrier concentration, channel length, and temperature that are associated with tunneling transport. We quantitatively describe the charge transport behavior of GNR heterojunction devices using a multi-barrier tunneling model. Performing chemical doping of GNR heterojunctions with alkali metal adatoms, we observe a highly superlinear modulation of the tunneling current in lieu of the conventional linear current modulation. The operation of our devices is based on modulation of bulk conductance through GNR heterojunctions, and not Schottky barriers. Our observations of tunneling conductance and chemical sensing using atomically precise GNR heterojunctions highlight their application potential.

## Results

### GNR heterojunction concept

**Fig. 1:**
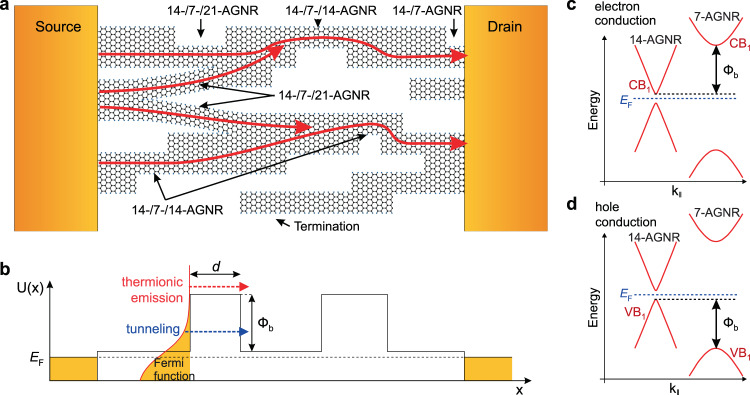
Electronic structure of GNR heterojunctions. **a** Schematic illustration of the aligned GNR heterojunctions integrated into the device. Lateral fusion of 7-AGNRs leads to the formation of quasi-metallic 14-AGNR and 21-AGNRs. When the source–drain contacts are fabricated, the remaining 7-AGNR segments act as tunneling barriers. Red arrows indicate different paths for charge transport. **b** Potential *U*(*x*) as a function of coordinate *x* between source and drain contacts of multiple 7-/14-AGNR heterojunctions. The Fermi level *E*_F_, the barrier height Φ_b_, and the barrier length *d* are indicated. The Fermi function shows the distribution of electrons in 14-AGNRs. Thermionic emission (red arrow) and tunneling (blue arrow) mechanisms are schematically shown. **c**, **d** Sketch of the electronic energy band dispersions of 7-AGNR and 14-AGNR. Momentum along the ribbon axis is denoted as **k**_∥_. The conduction and valence band edges are CB_1_ and VB_1_, respectively, and determine the value of Φ_b_.

Densely aligned, parallel 7-AGNRs can be grown on a stepped Au(788) crystal^20,21^. Thermally activated lateral fusion leads to the formation of narrow-bandgap (quasi-metallic) 14-AGNR segments as illustrated in Fig. 1a. Wider GNRs with *N* = 21, 28, and so forth are less abundant, and similarly to 14-AGNRs are also quasi-metallic^12^. GNR segments of different widths form an aligned array of GNR heterojunctions, where quasi-metallic GNRs are connected by wide-bandgap 7-AGNR segments, for example, 14-/7-/14-AGNR or 14-/7-/21-AGNR. Parallel sequences of heterojunctions may also join one another to form “Y-junctions,” e.g., a 14-AGNR may split into two 7-AGNRs that are each in turn fused with two neighboring GNRs. Charge transport can be probed by transferring such a GNR heterojunction array to an insulating substrate and depositing metallic electrodes to form source and drain contacts. The wide-bandgap 7-AGNR segments act as energy barriers. GNR heterojunctions form a variety of conducting paths, which may also intersect with one another. Charge carriers traverse a multiple barrier potential profile along their path from source to drain (Fig. 1b). There are two primary mechanisms of charge transport from one quasi-metallic segment to another: tunneling through the 7-AGNR barrier and thermionic emission over the barrier (Fig. 1b). The tunneling and thermionic currents depend on temperature (with the temperature dependence of the latter much stronger than the former), on the applied electric field, and on the barrier shape. The barrier length *d* is the length of the 7-AGNR segment. For electron (hole) injection from the 14-AGNR segment into the 7-AGNR segment, the barrier height Φ_b_ is given by the conduction (valence) band offset, i.e., the difference between the first conduction (valence) sub-bands of the 7- and 14-AGNR (Fig. 1c, d). Due to the large bandgap of 7-AGNRs, the difference in Φ_b_ between the 7-/14-AGNR and the 7-/21-AGNR junctions can be neglected. GNR fusion can also produce non-lateral heterojunctions, such as heterojunctions between the lateral armchair edge and the terminal zigzag edge of an AGNR. These non-lateral heterojunctions may host localized midgap edge states^22^, which alter the effective barrier. However, as we will see from the STM data presented below, our GNRs are aligned, and therefore the density of such junctions is minimized. Moreover, the edge state wave function falls off rapidly in the GNR bulk^23^, therefore we do not expect a substantial change to the barrier transparency, which is determined by *d* and Φ_b_. In Supplementary Note 1 (Supplementary Fig. 2), we also show scanning tunneling spectroscopy (STS) measurements of our GNR heterojunction system, which suggest the similarity of the local density of states of 7-AGNR segments regardless of the configuration of GNR heterojunctions.

### Synthesis and characterization of aligned GNR heterojunctions

**Fig. 2:**
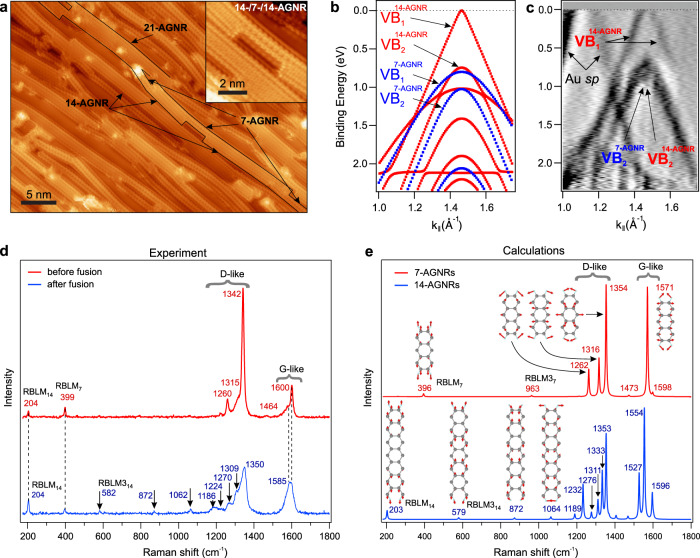
Experimental characterization of the aligned GNR heterojunctions on Au(788). **a** STM topographic image of fused 7-AGNRs (sample bias *V*_s_ = −1.3 V, tunneling current *I*_t_ = 1.8 nA). The black lines outline one possible conducting path through the GNR heterojunctions: quasi-metallic 14- and 21-AGNRs connected by 7-AGNR segments. The inset shows an example of a typical 14-/7-/14-AGNR heterojunction with a short (~3 nm) 7-AGNR segment. See also Supplementary Note 1. **b** Calculated electronic band structure of 7-AGNRs (blue) and 14-AGNRs (red) shown in the second Brillouin zone of GNRs, where the ARPES scans were acquired. The first and the second valence sub-bands of 14-AGNRs (7-AGNRs) are labeled as VB${\,}_{1}^{14\mbox{-}{\rm{AGNR}}}$ (VB${\,}_{1}^{7\mbox{-}{\rm{AGNR}}}$) and VB${\,}_{2}^{14\mbox{-}{\rm{AGNR}}}$ (VB${\,}_{2}^{7\mbox{-}{\rm{AGNR}}}$), respectively. The valence band maxima in the calculations are aligned to the ARPES data. **c** Second derivative with respect to momentum of the ARPES scan (to enhance the contrast) of fused GNRs on Au(788) measured along the GNR axis (**k**_∥_) with fixed in-plane momentum perpendicular to the axis (**k**_⊥_)^37^. To maximize the photoemission intensity from VB${\,}_{1}^{14\mbox{-}{\rm{AGNR}}}$, we used **k**_⊥_ = 1.1 Å^−1^. At this **k**_⊥_, the intensities from VB${\,}_{2}^{14\mbox{-}{\rm{AGNR}}}$ and VB${\,}_{2}^{7\mbox{-}{\rm{AGNR}}}$ overlap (Supplementary Note 2). The Au *sp* bands that are from the substrate are also indicated. **d** UHV Raman spectra (300 K, 633 nm) of GNRs on Au(788) before and after fusion. The frequencies of the respective Raman peaks are indicated (values in cm^−1^). **e** Calculated Raman spectra of 7-AGNR and 14-AGNR. The structure of 7-AGNR and 14-AGNR unit cells with the eigenvectors of selected phonon modes are shown (see also Supplementary Note 4). The arrows indicate the atomic displacement. The RBLM3_7_ and RBLM3_14_ are the third overtones of RBLM_7_ and RBLM_14_, respectively.

STM studies reveal that the lateral fusion (see “Methods”) of densely aligned 7-AGNRs on an Au(788) crystal leads to the formation of numerous 14-AGNRs and wider GNRs. (Fig. 2a and Supplementary Note 1). The fused GNRs have a length of the order of tens of nm, and many of them are connected by short (several nm) 7-AGNR segments. Together, these form well-aligned paths for charge transport. The linear density of 7-AGNR tunneling barriers is about one per 20 nm, and the average barrier length is about 4 nm (Fig. 2a and Supplementary Fig. 1). We characterize fused GNRs by ARPES and ultra-high vacuum (UHV) Raman spectroscopy^21^. Upon fusion of aligned 7-AGNRs, we observe new features in the ARPES spectra (Supplementary Note 2). A linearly dispersing energy band is observed, consistent with calculations (Fig. 2b, c and “Methods”). Based on the peculiar variation of the photoemission intensity for GNRs in momentum space (Supplementary Note 2), we attribute this band to the first valence sub-band of 14-AGNRs, labeled VB${\,}_{1}^{14\mbox{-}{\rm{AGNR}}}$ hereinafter. The apex of VB${\,}_{1}^{14\mbox{-}{\rm{AGNR}}}$ is touching the Fermi level (*E*_F_), which is in line with the observed Fermi level pinning in GNRs on an Au substrate^24^. UHV Raman spectra before and after the fusion were measured in situ (Fig. 2d) and are compared to the calculations (see “Methods”) for 7-AGNRs and 14-AGNRs (Fig. 2e). The initial Raman spectrum consists primarily of 7-AGNR-derived modes. Upon fusion, we observe changes in the regions of the G-like and D-like modes (whose atomic displacements resemble the G and D modes in graphene) and the appearance of well-separated peaks in the low-frequency region. We observe radial breathing-like modes at 399 cm^−1^ for 7-AGNRs (RBLM_7_) and at 204 cm^−1^ for 14-AGNRs (RBLM_14_)^25,26^. The frequencies of all peaks that appear after fusion are in excellent agreement with the calculations for 14-AGNRs. During GNR fusion, we have monitored the UHV Raman spectrum, which allowed us to optimize the process for maximum 14-AGNR peak intensities (Supplementary Note 4). STM, ARPES, and Raman spectroscopic measurements consistently show that the fused sample is a monolayer of aligned multiple heterojunctions of quasi-metallic GNRs and wide-bandgap 7-AGNR segments.

### Charge transport characterization

**Fig. 3:**
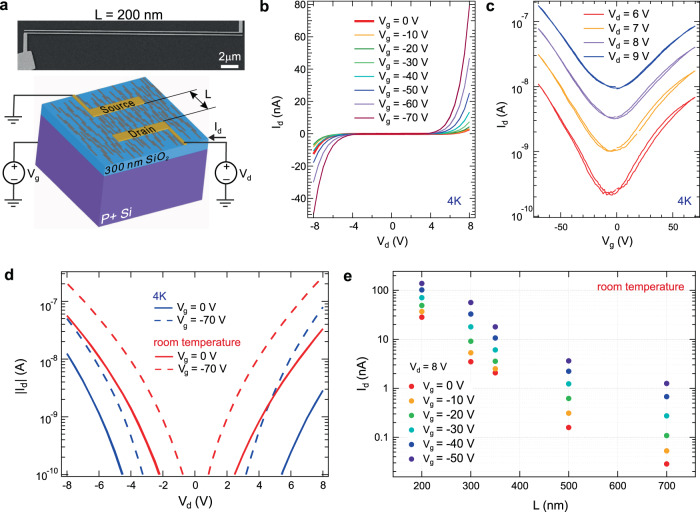
Charge transport characterization of the transferred GNR heterojunctions. **a** Top: scanning electron microscopy image of the device with the channel length *L* = 200 nm and the channel width *W* = 25 μm. Bottom: sketch of aligned GNR heterojunctions on SiO_2_/Si in a FET geometry with source, drain, and gate contacts. **b**–**d** Transport characteristics of the device with *L* = 200 nm. **b** *I*_d_–*V*_d_ curves at different *V*_g_, and **c** *I*_d_ versus *V*_g_ at different *V*_d_ at 4 K. **d** *I*_d_–*V*_d_ curves in log scale at 4 K (blue) and room temperature (red) at *V*_g_ = 0 and −70 V. **e** Channel length dependence of the *I*_d_ at *V*_d_ = 8 V and different *V*_g_ at room temperature.

For the transport measurements, a film of aligned GNR heterojunctions was transferred to a doped Si wafer with 300 nm SiO_2_ using electrochemical delamination^25^ to fabricate back-gated FETs. The GNR orientation and structural quality of the transferred sample were confirmed by polarized Raman measurements (Supplementary Note 4). Electrical contacts to the film were fabricated by electron-beam lithography. In the FET devices (Fig. 3a), the drain current *I*_d_ was measured as a function of the drain voltage *V*_d_ and the back-gate voltage *V*_g_. We fabricated devices with different channel lengths *L* and a fixed channel width *W* (25 μm). The GNR heterojunctions were aligned along the channel between the source and drain contacts. After device fabrication, each sample was mounted on a sample holder that enables charge transport measurements to be carried out in UHV.

We observe nonlinear *I*_d_–*V*_d_ behavior and a clear *V*_g_ dependence (Fig. 3b). A strong field effect is observed for both electron (*V*_g_ > 0) and hole (*V*_g_ < 0) conduction, demonstrating bipolar operation (Fig. 3c). The current *I*_d_ is modulated by *V*_g_ by two orders of magnitude, with higher hole conduction than electron conduction. We extract the field-effect mobility using the direct transconductance method (DTM): *μ*_DTM_ = 6.0 × 10^−5^ cm^2^/Vs at *V*_d_ = 6 V and *μ*_DTM_ = 8.3 × 10^−4^ cm^2^/Vs at *V*_d_ = 9 V (see “Methods”). Such low values of *μ*_DTM_ cannot be explained by conventional band transport and are more typically associated with hopping transport, which occurs due to charge carrier transitions between localized states^27–30^. However, the temperature dependence of *I*_d_ in our devices does not agree with a variable-range hopping model (Supplementary Note 5 and Supplementary Fig. 7). Our devices have weak temperature dependence. Increasing the temperature from 4 K to room temperature increases the current by less than one order of magnitude (Fig. 3d, Supplementary Note 5, and Supplementary Fig. 6). Therefore, we exclude hopping transport, as well as the thermionic emission over the barrier as the conduction mechanisms in our system. In Supplementary Note 6, we also compare the temperature dependence of conduction in our aligned heterojunctions to the model of nuclear tunneling of polarons, which was recently used by Richter et al. to interpret the charge transport in a network of narrow-bandgap 5-AGNRs^31^. While the fit parameters in the master equation used by Richter et al. for 5-AGNRs look very reasonable, the fit parameters needed to describe our data by the same master equation are rather unrealistic. Therefore, we cannot claim the polaron conduction between quasi-metallic AGNRs as a dominant transport mechanism in our system. Note that the structure of our system, consisting of alternating wide-bandgap and quasi-metallic GNR segments, is different from those of the 5-AGNR network, where narrow-bandgap GNRs are densely packed. Quantum mechanical tunneling through potential barriers is compatible with the dependencies observed in our GNR heterojunctions^32^. Further evidence that tunneling through 7-AGNR segments governs the transport is the channel length dependence of conduction in our FETs. We observe an exponential drop in *I*_d_ with increasing *L* (Fig. 3e). The exponential trend in *I*_d_ differs from the 1/*L* scaling of conductance of an Ohmic conductor. If Schottky barriers would dominate the charge transport, all the applied *V*_d_ would drop near the contacts. In this situation, *I*_d_–*V*_d_ characteristics of devices with different *L* should be the same, up to multiplication of the current by a constant factor that reflects different numbers of conducting paths in different devices. As a consequence, the slopes of *I*_d_–*V*_d_ curves in semilogarithmic plots (log(*I*_d_) versus *V*_d_) should be independent of the channel length. However, in our data this is not the case, as we will show later. In single semiconducting GNR devices with short contact separation, Schottky barriers led to nonlinear *I*_d_–*V*_d_ characteristics^19^. Our devices have a contact separation *L* ≥ 200 nm, and several 7-AGNR barriers are traversed by charge carriers between the source and drain. Therefore, these tunneling barriers are expected to dominate device resistance. In the “Methods,” we quantitatively compare the impact of heterojunction barriers and of Schottky barriers on the total device resistance and find that the contribution of the latter is negligible. Finally, we note that the statistical variation from device to device is insignificant as compared to the observed trend with length *L*. Different devices of the same *L* show a narrow distribution of conductance (Supplementary Note 5 and Supplementary Figs. 8 and 9). Consequently, conduction is not governed by a small number of conducting paths, as is the case in molecule-based systems^33^. Rather, the number of contributing paths is sufficiently high to be considered a true ensemble measurement of conduction through AGNRs.

### Tunneling barrier analysis of transport measurements

**Fig. 4:**
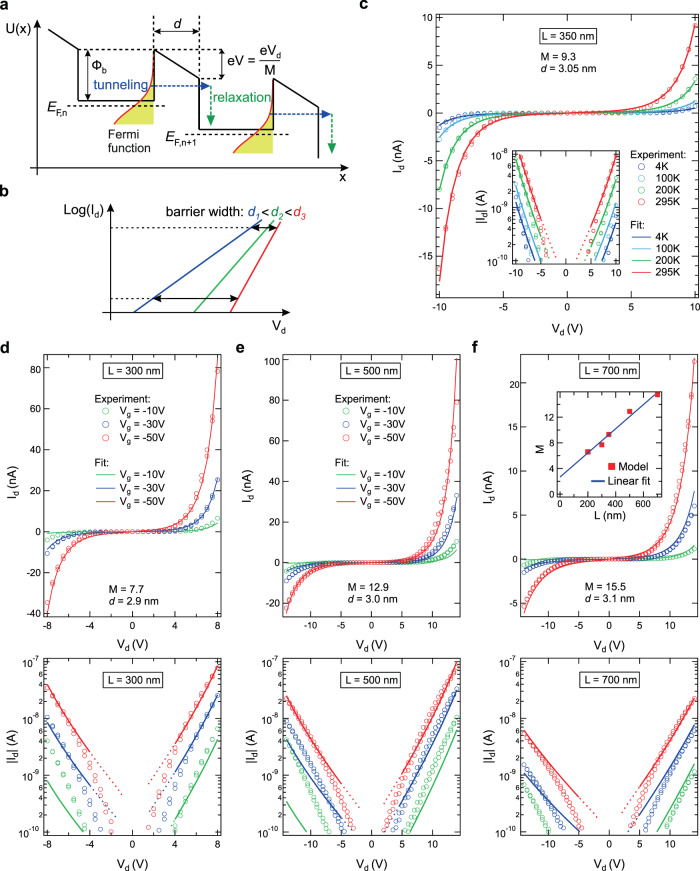
Comparison of experimental and calculated *I*_d_–*V*_d_ curves. **a** The energy diagram along one conducting channel of GNR heterojunctions. The tunneling through a barrier (blue arrows) followed by relaxation of the carrier (green arrows) is illustrated. The Fermi level of the *n*th barrier is indicated as *E*_F,*n*_. The energy drop across one barrier, equal to *eV* = *eV*_d_/*M* (where *e* is the electron charge and *M* is the number of barriers in the channel), is indicated along with the barrier height Φ_b_ and the barrier length *d*. **b** Sketch of ${\mathrm{log}}\,({I}_{{\rm{d}}})$ versus *V*_d_ characteristics of three different barriers. At high *I*_d_, the voltage drops *V*_d_ across the barriers assume a narrower distribution (indicated by horizontal arrows). **c** Experimental (empty circles) and calculated (solid lines) *I*_d_–*V*_d_ curves of the 350 nm channel device at different temperatures between 4 and 295 K at *V*_g_ = 0. The inset shows the *I*_d_–*V*_d_ curves in a log scale. **d**–**f** Experimental (empty circles) and calculated (solid lines) *I*_d_–*V*_d_ curves for devices with *L* = 300, 500, and 700 nm at *V*_g_ = −10, −30, and −50 V at 295 K in linear (top) and log (bottom) scales. The inset in **f** shows the *M* versus *L* dependence and its linear fit.

We model our system as a set of parallel conducting quasi-metallic 14-AGNRs that are connected in series by 7-AGNR tunneling barriers (Fig. 4a). A voltage *V*_d_ across the contacts leads to trapezoidal barrier potential profiles, corresponding to the development of electric field and potential drops across the semiconducting barrier segments. The potential drop per semiconducting segment is *V* = *V*_d_/*M* where *M* is the number of junctions between the contacts. The tunneling of a charge carrier through the barriers is considered to be sequential. A charge carrier entering a 14-AGNR segment following a tunneling event through a 7-AGNR barrier undergoes rapid inelastic scattering through emission of optical phonons. In the related material of carbon nanotubes (CNTs), charge carriers are scattered by optical phonon emission on length scales estimated to be as short as 10 nm^34^. To describe the *I*_d_–*V*_d_ characteristics, we use the Wentzel–Kramers–Brillouin (WKB) approximation^35,36^. Equation (4) of ref. ^36^ expresses the tunneling current *I* as a function of the voltage *V* across a trapezoidal barrier as

1 $$I(V)=\frac{2e}{h}\mathop{\int}\nolimits_{-\infty }^{\infty }P(E)\left[f(E)-f(E+eV)\right]{\rm{d}}E.$$

Here *e* and $h$ are the electron charge and Planck’s constant, respectively. The Fermi distribution function is given by

2 $$f(E)=\frac{1}{1+\exp \left(\frac{E-{E}_{{\rm{F}}}}{kT}\right)},$$

where *E*_F_ depends on *V*_g_ and *V*_d_ as ${E}_{{\rm{F}}}({V}_{{\rm{g}}},{V}_{{\rm{d}}})={E}_{{\rm{F}}}^{0}(T)+\alpha (T)({V}_{{\rm{g}}}+\beta {V}_{{\rm{d}}})$. Here ${E}_{{\rm{F}}}^{0}$, *α*, and *β* are phenomenological fitting parameters. The parameter *α* accounts for the modulation of channel potential with *V*_g_, and *αβ* accounts for the modulation of channel potential by *V*_d_ (see “Methods”). The quantity ${E}_{{\rm{F}}}^{0}(T)$ accounts for the temperature dependence of *E*_F_. *P*(*E*) in Eq. (1) is the tunneling probability through the barrier of length *d* and is given by the following expression^36^:

3 $$P(E)=A\exp \left(-\frac{2}{\hbar }\mathop{\int}\nolimits_{0}^{d}\sqrt{2m[\varphi (x,V)-E]}{\rm{d}}x\right).$$

Here *φ*(*x*,*V*) = Φ_b_ + (*x*/*d*) ⋅ (−*eV*) is the barrier height as a function of the coordinate *x*, Φ_b_ denotes the barrier height at *x* = 0, and *m* is the effective mass inside the barrier which we take from ARPES data^37^. The prefactor *A* is proportional to the number of parallel 1D channels across the source–drain contacts and it was not allowed to vary significantly for all devices and it was kept constant for the *L*-dependent measurements shown below. The barrier height Φ_b_ is fixed at a constant value of Φ_b_ = 1.35 eV for all fits throughout our work (see “Methods” for determination of Φ_b_ for GNR heterojunctions on SiO_2_). The real GNR heterojunction system consists of several barriers in sequence where the tunneling current is limited by the longest and most opaque barrier. For simplicity, our model ignores the distribution of *d* within one device and assumes one effective value of *d*. Figure 4b depicts a sketch of the *I*_d_–*V*_d_ relations for three barriers with different *d* indicating that the voltage drop across each barrier becomes closer to each other at high *I*_d_. Thus, our model is more accurate in the high *I*_d_–*V*_d_ regime. At low *I*_d_ and *V*_d_, the effects of disorder including the distribution of *d* and *M*, trap states, and inhomogeneous surface potential are expected to play a more important role.

Figure 4c depicts the temperature dependence of the experimental and modeled *I*_d_–*V*_d_ characteristics of a *L* = 350 nm device for *T* = 4, 100, 200, and 295 K. The experimental data for *L* = 300, 500, and 700 nm devices at different *V*_g_ were fitted by restricting the number of tunneling barriers *M* to be proportional to the channel length *L* (Fig. 4d–f). To estimate the role of contacts, we plot *M* as a function *L* (Fig. 4f, inset). By analogy with the conventional transfer length method used for Ohmic conductors, we interpret the linear extrapolation of this dependence to *L* = 0 as the effective number of tunneling barriers at the contacts. This yields 2.6 barriers in total for two contacts, or 1.3 effective barriers per contact. Therefore, our model corroborates our conclusion about the relative insignificance of contact Schottky barriers in our experiments. In all FETs, the fit yields practically identical values of tunneling barrier length *d* ≈ 3 nm. The semilogarithmic plots are shown in the lower panels in Fig. 4d–f. One can see that the slope of the *I*_d_–*V*_d_ curves in the semilogarithmic plots decreases with increasing *L*. This is because of the increase in *M* and indicates that *V*_d_ drops not only at the contacts but also inside the channel. The semilogarithmic plots reveal a generally worse agreement of the model fit with experiment at small *I*_d_ and *V*_d_ as discussed in the context of Fig. 4b. Our model accounts for the experimentally observed charge transport behavior of GNR heterojunctions over a wide range of experimental conditions, including temperature, *V*_g_, and length dependence of *I*_d_–*V*_d_ characteristics, using a set of only six fit parameters (*A*, *d*, *M*, ${E}_{f}^{0}(T)$, *α*(*T*), and *β*).

One might question on whether the 7/14- or 7/21-AGNR heterojunctions are truly what is the most important aspect limiting charge transport, because the conduction path is rather complicated. It includes connections between adjacent branches, and the distribution of current and voltage across junctions varies continuously upon applied bias due to the nonlinear nature of the elementary junction conductance. However, our STM data show that our network is composed out of aligned atomically precise wide-bandgap/quasi-metallic AGNR heterojunctions of well-defined types. Thus, each charge traverses a set of well-defined elementary junctions. Although we cannot be certain about the particular structure of the conducting path, the assumption that each path always traverses a set of well-defined junctions is realistic. In our model, we assume that we have *M* such junctions in series. Agreement between the simulation results and the experimental data cannot probably serve as unambiguous proof for this simplified model. This, however, does not exclude that the gate-tunable tunneling conductance across the 7-AGNR barrier is at the heart of the observed transport properties.

### Tunneling current modulation by adsorbates

**Fig. 5:**
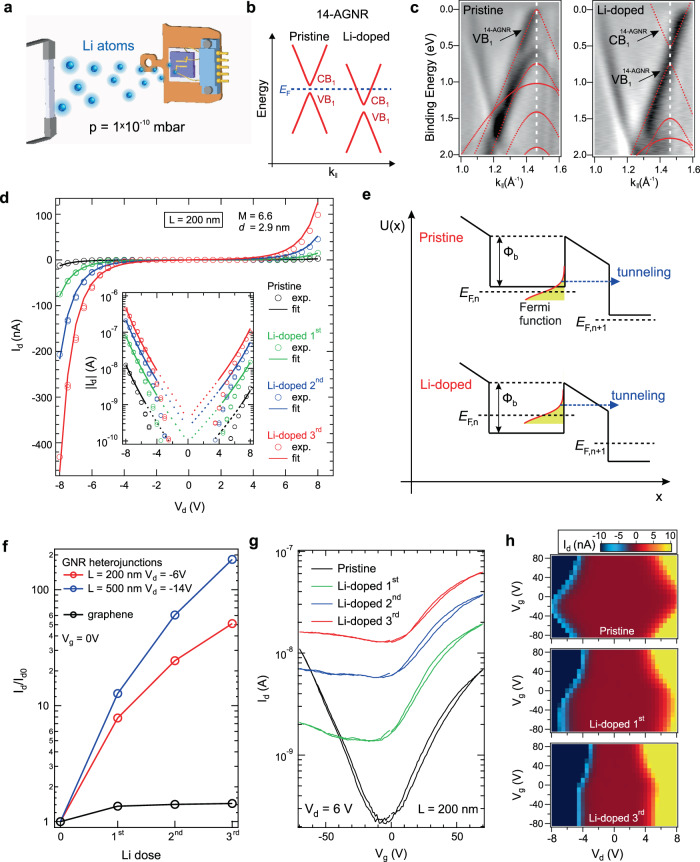
Band structure and charge transport of Li-doped GNR heterojunctions. **a** Sketch of the experimental set-up containing a Li source and the GNR heterojunction FET mounted on a UHV compatible sample holder. **b** Sketch of the band structure changes of 14-AGNRs upon Li doping. **c** Second derivatives of ARPES scans by momentum of the GNR heterojunctions on Au(788) before (left) and after (right) Li deposition (~1 Å) at **k**_⊥_ = 0.71 Å^−1^ (Supplementary Note 3). Dashed vertical white line denotes the center of the second Brillouin zone of AGNRs. Red dashed lines are the calculated electronic band structure, aligned in energy to the ARPES data. **d** *I*_d_–*V*_d_ characteristics before (pristine sample) and after deposition of three identical Li doses (~0.1 Å each) in linear and log (inset) scales for the *L* = 200 nm device. Experimental points (exp.) are shown by circles, the fit is indicated by solid lines. **e** Schematic illustration of the potential profile *U*(*x*) across a tunneling barrier for pristine and Li-doped GNR heterojunctions. **f** The ratio (log scale) of current after Li doping *I*_d_ to the current in pristine sample *I*_d0_ for three Li doses for the *L* = 200 nm device at *V*_d_ = − 6 V, for the *L* = 500 nm device at *V*_d_ = −14 V, and for the graphene FET (Supplementary Note 8), all at *V*_g_ = 0 V. **g** *I*_d_–*V*_g_ characteristics of the *L* = 200 nm device at *V*_d_ = 6 V for three Li doses. **h** Color maps of the dependence of *I*_d_ on *V*_g_ and *V*_d_ for the pristine and Li-doped *L* = 200 nm device. All transport measurements were performed at 4 K.

We performed in situ doping of our devices by Li adatoms in a UHV system (Fig. 5a), observing a strong modulation of the transport properties of the GNR heterojunctions. Upon chemical doping by Li, the *E*_F_ shifts deeply into the conduction band of quasi-metallic GNRs as schematically shown in Fig. 5b. ARPES spectra of fused GNRs on Au(788) reveal the shift of VB${\,}_{1}^{14\mbox{-}{\rm{AGNR}}}$ relative to *E*_F_ by ~0.7 eV after deposition of ~1 Å of Li, visualizing the partially occupied CB${\,}_{1}^{14\mbox{-}{\rm{AGNR}}}$ (Fig. 5c and Supplementary Note 3). Compared to 14-AGNRs, achieving degenerate electron doping of wide-bandgap 7-AGNRs requires a much larger Li coverage^21^. The *I*_d_–*V*_d_ characteristics reveal a dramatic increase in channel current upon Li doping performed in three consecutive steps of ~0.1 Å each (Fig. 5d and Supplementary Fig. 11). The *I*_d_–*V*_d_ curves of Li-doped GNR heterojunctions are accurately reproduced by our tunneling barrier model (Fig. 5d). The surface doping of GNR heterojunctions by Li adatoms shifts *E*_F_ and effectively reduces the barrier height to Φ_b_ − *E*_F_ (Fig. 5e). In the model calculations, *M* and *d* were held constant. The fit values of *E*_F_ for the sample in its pristine state and after Li doses 1–3 were 4, 25, 63, and 103 meV relative to CB${\,}_{1}^{14\mbox{-}{\rm{AGNR}}}$, respectively. These values are in good agreement with the shifts inferred by comparison with ARPES (Supplementary Note 7). Our model is also accurately reproducing the *I*_d_–*V*_d_ characteristics of the Li-doped *L* = 500 nm device (Supplementary Note 7).

The channel current through a band conductor scales approximately linearly with charge carrier density. Surface doping of graphene by alkali metal adatoms leads to a modest increase in channel current as a result of the combined effects of increased carrier density and reduced mobility due to charged impurity scattering^38^. In contrast, in an ideal system the tunneling current through a GNR heterojunction is exponentially dependent on the tunneling barrier height. As a consequence, upon alkali metal doping we observe an increase in the current ratios of the doped and pristine samples (*I*_d_/*I*_d0_) by a factor 50 for the *L* = 200 nm device operated at *V*_d_ = −6 V and by a factor 180 for the *L* = 500 nm device operated at *V*_d_ = −14 V (Fig. 5f). According to our tunneling model, the difference in current modulation for the two devices is mostly related to the voltage drops across one heterojunction (*V*_d_/*M*) and to the slightly different values of *E*_F_ (Supplementary Note 7). We also performed identical adsorption experiments using a graphene FET (Supplementary Note 8). The current in graphene increases only by a factor <2 after deposition of identical amounts of Li (Fig. 5f). The adsorbate-induced current modulation in the GNR heterojunction FETs is highly nonlinear in Li dose. The operation of our devices based on modulation of the channel conductance, and therefore GNR heterojunction FETs, differ from the CNT Schottky barrier transistors, in which adsorbates modulate electron tunneling only at the contact^39^. To confirm the potential application of GNR heterojunctions for tunneling devices, we analyze the channel referenced sub-threshold swing *S*_ref_, which is the Fermi level change (known as the channel potential change) required to modulate the drain current by one decade: ${S}_{{\rm{ref}}}=e^{-1}\partial {E}_{{\rm{F}}}/\partial {{\rm{log}}}_{10}({I}_{{\rm{d}}})$. At room temperature for the *L* = 200 nm GNR heterojunction FET (Fig. 3d), we obtain *S*_ref_ = 34 mV/dec at *V*_d_ = 8 V (see “Methods”). This value is smaller than the fundamental thermionic limit of ${\rm{ln}}(10){k}_{{\rm{B}}}T/e$ = 60 mV/dec in metal oxide semiconductor FETs. GNR heterojunctions can thus be applied as a sensor that unites the steep slope current scaling of TFETs with an accessible surface for adsorption. We find a sensitivity to adsorbates of *s* = ∂*I*_d_/∂*q* = 2.4 × 10^−12^ nA cm^2^, where *q* is the Li dose per unit area (see “Methods”). With the observed current noise of Δ*i*_d_ ≈ 2 pA, the corresponding sensor resolution to Li dose is *r* = Δ*i*_d_/*s* ≈ 8 × 10^8^ cm^−2^, equivalent to 8 Li atoms per square micron, or 1 ppm of a monolayer of Li.

The adsorption of Li modifies the field effect of GNR heterostructures in a non-trivial fashion (Fig. 5g). We observe the loss of bipolar field effect, wherein electron conduction can be modulated by *V*_g_ while modulation of hole conduction is strongly suppressed. As illustrated in Fig. 5h, in the pristine system, a “diamond”-like shape emerges in the plot of *I*_d_ versus *V*_g_ and *V*_d_ corresponding to bipolar gate modulation of both electron and hole currents. The “diamond” is reminiscent of that emerging in Coulomb blockade^40^ but is here a direct consequence of the gate modulation of tunneling conduction. Upon Li deposition, *I*_d_ no longer exhibits a field effect for *V*_g_ < 0. For comparison, graphene exhibits a bipolar field effect with a shifted neutrality point after the first Li dose (Supplementary Fig. 12), in good agreement with previous work in the low-doping regime^38^. At higher Li doses, only electron conduction can be modulated by gate voltage (Supplementary Fig. 12), in agreement with previous work in the high-doping regime where the mismatch in density of states within bulk and contact graphene regions imparts asymmetry to the field effect^41^. The asymmetric field effect in heavily doped GNR heterojunction FETs may thus originate from several mechanisms. In common with graphene, a shift in density of states between bulk and contact regions is expected. Furthermore, Li adsorption can occur on the GNR surfaces, GNR edges, and on the exposed SiO_2_ surface, contributing to a more complex environment for charge exchange than graphene. Further work to understand the microscopic mechanisms of field effect in heavily doped GNR heterojunctions is required.

We note that alkali metals are strong electron donors and hence induce a large current modulation in our devices. However, the working principle of sensing any adsorbate in this kind of sensor is charge transfer. Some gases even have a large charge transfer of one elementary charge per adsorbed molecule^42^. We thus expect that GNR heterojunction-based sensors can also effectively detect other adsorbates, provided their charge transfer is sufficiently large. To achieve a scaled, single junction device with steep sub-threshold swing, the minimal GNR structure required for tunneling transport must be identified and successfully integrated in a transistor structure with high gate efficiency^43^.

## Discussion

In summary, we have synthesized a monolayer film of aligned atomically precise heterojunctions of wide-bandgap and quasi-metallic GNRs on Au(788) and comprehensively characterized them by STM, ARPES, and Raman spectroscopy. After the alignment-preserving transfer of the film onto a SiO_2_/Si substrate, we have measured charge transport along the GNR heterojunctions in a back-gated FET geometry. The characteristic dependencies of the current on drain and gate voltages, temperature, and channel length indicate that charge transport proceeds by quantum mechanical tunneling through the wide-bandgap 7-AGNR segments in the GNR heterojunctions. The experimental transport data are in agreement with computations (WKB approximation) of the tunneling current through multiple tilted barriers. Our model is able to describe all observed experimental current–voltage characteristics in the high bias regime using a minimal set of fit parameters, including the effective barrier height, the junction length, and the number of junctions. In our samples, the uniformity achieved in the atomically precise GNR heterojunctions and the degree of GNR alignment is sufficient to observe tunneling transport in the GNR heterojunction network. This is one of the key experimental findings of our work. We have demonstrated that the adsorption of atoms on the GNR heterojunction surface strongly modulates the tunneling conductance by charge transfer doping. We employed the steep slope response of GNR heterojunction-based tunnel FETs with their accessible surface area to realize a nanoelectronic sensor with a giant sensitivity to adsorbates. Our results are important not only for applications of bottom–up synthesized carbon nanomaterials but also for the wider nanoscience community that applies low-dimensional materials in new device concepts.

## Methods

### Growth of GNR heterojunctions

The synthesis of aligned GNR heterojunctions for our tunneling devices consists of the following steps: (1) deposition of approximately one monolayer coverage of 10,10-dibromo-9,9 bianthracene molecules on a clean Au(788) surface (prepared by standard Ar^+^ sputtering and annealing cycles) in UHV; (2) dehalogenation of the precursor molecules and assembly of the aligned polymer chains by annealing at ~200 °C for 10 min; (3) cyclodehydrogenation and lateral fusion at ~410 °C for 180 min. The first two steps are identical to the synthesis of densely aligned 7-AGNRs on Au(788)^20,25,37^ while the third step is performed at higher temperature and longer annealing time. The annealing temperature and time were optimized to get a maximum amount of 14-AGNRs using in situ studies with UHV Raman spectroscopy (Supplementary Note 4).

### STM measurements

STM imaging of an in situ prepared GNR heterojunctions sample was done at room temperature and in UHV (base pressure was 4 × 10^−11^ mbar) in the Athene STM chamber in Cologne. The STM images were processed (background subtraction and contrast adjustment) using the WSxM software^44^.

### ARPES experiments

During ARPES experiments, the base pressure was <2 × 10^−10^ mbar. In all experiments, the aligned GNR heterojunctions were oriented along the slit of analyzer and linearly horizontally polarized light was used. The ARPES measurements shown in Fig. 2c and Supplementary Figs. 3a–e have been performed at Hiroshima Synchrotron Radiation Center (beamline BL-9A/B) using a horizontal analyzer slit and the tilt angle of manipulator to tune **k**_⊥_ at 10 K with the photon energy of 25 eV. The GNR synthesis and fusion were performed in situ. ARPES experiments on Li doping (Fig. 5c and Supplementary Fig. 4) and the ARPES data shown in Supplementary Figs. 3f–j have been performed at HZB BESSY II (UE112-PGM2 beamline, 1^2^-ARPES end-station) using a vertical analyzer slit and the polar angle of manipulator to tune **k**_⊥_ at room temperature with the photon energy of 45 eV. The sample was synthesized at the University of Cologne and preliminary checked in situ by UHV Raman spectroscopy. Then the sample was transferred to the ARPES end-station in a suitcase filled by Ar. Li doping was performed in situ using a SAES getter. The amount of deposited Li, estimated by a quartz crystal microbalance sensor, was about 1 Å.

### Energy band structure calculations and ARPES intensity simulations

Density functional theory (DFT) calculations of the band structure of 7-AGNRs and 14-AGNRs were carried out using the FPLO-14.00-48 code (improved version of the original FPLO code by Koepernik and Eschrig)^45^ utilizing the generalized gradient approximation (GGA) to the exchange–correlation potential. The GNRs were assumed freestanding and hydrogen-terminated. A **k**-point grid of 12 × 1 × 1 was used to sample the Brillouin zone. Atomic positions were relaxed until the forces on each atom were <10^−2^ eV/Å. The calculated electronic bands of 7-AGNRs and 14-AGNRs that are shown in Fig. 2b were shifted in energy to match the experimental VB_1_. The photoemission intensity that is shown in Supplementary Fig. 3 was calculated using the dipole approximation for the matrix element with a plane wave as a final state^37,46^.

### Raman spectroscopy measurements

All Raman measurements presented in this work were performed in the back-scattering geometry using a Renishaw inVia set-up with a 633-nm laser at room temperature. UHV Raman studies shown in Fig. 2 were performed in the same set-up where the GNR heterojunctions were synthesized^21^. In the UHV studies, the laser was polarized along the GNR alignment direction (*z*), and the collected Raman signal was a sum over the GNR plane (*zz* and *zy*). UHV Raman data were acquired using a ×50 long-working distance objective with a numerical aperture of 0.4 and the laser power of 7 mW. This laser power does not affect the sample quality due to the UHV conditions. Polarized Raman measurements of the GNR heterojunctions transferred onto the SiO_2_/Si substrate, shown in Supplementary Fig. 5d, were performed in ambient conditions using a ×50 objective and 0.4 mW laser power. The low wavenumber data, shown in Supplementary Fig. 5c, were acquired with the laser polarized along the alignment direction of GNR heterojunctions using a notch filter and 0.8 mW laser power.

### Calculation of Raman spectra

Theoretical modeling of the Raman spectra has been performed in the framework of DFT. All computations were carried out within the AIMPRO DFT package^47,48^. Perdew–Burke–Ernzerhof GGA^49^ has been used as exchange–correlation functional. The action of core electrons was modeled using Hartwigsen–Goedecker–Hutter pseudopotentials^50^ and the electron wave functions have been expanded into a basis set of Gaussian orbitals. The carbon atom basis consisted of 38 *s*-, *p*-, and *d*-type functions, whereas for hydrogen a total of 12 *s*- and *p*-type functions were used. A **k**-point grid with the resolution of 2*π* × 0.01 Å^−1^ along the periodic direction of the GNR has been employed for the self-consistent field cycle. After geometry optimization, phonon eigenvalues and eigenvectors from the Brillouin zone center have been calculated with the use of the finite-displacement method. The Raman tensor has been calculated by considering finite atomic displacements along the phonon eigenvectors and by calculating the frequency-dependent dielectric tensor for each eigenmode. For the dielectric tensor calculation, a fine **k**-point grid with a resolution of 2*π* × 0.0006 Å^−1^ along the GNR axis has been used. A scissor shift for the bandgap has been applied in order to bring the calculated gap values in closer agreement with experiment (2.1 eV for 7-AGNR and 0.25 eV for 14-AGNR). The Raman intensity for the *j*-th mode at excitation energy *E*_exc_ was obtained as $I\propto \frac{{n}_{j}+1}{{\omega }_{j}}| {{\bf{e}}}_{{\rm{i}}}\cdot {{\bf{R}}}_{j}({E}_{{\rm{exc}}})\cdot {{\bf{e}}}_{{\rm{s}}}^{{\rm{T}}}{| }^{2}$, where *ω*_*j*_ is the phonon frequency, ${n}_{j}={({e}^{\hslash {\omega }_{j}/{k}_{{\rm{B}}}T}-1)}^{-1}$ is the Bose–Einstein distribution function at *T* = 300 K, **e**_i_ and **e**_s_ are electric polarization vectors of the incident and scattered optical fields, respectively, and **R**_*j*_(*E*_exc_) is the Raman tensor of phonon mode *j* at excitation energy *E*_exc_. In the experimental scattering geometry, the incident light is polarized along the periodic GNR axis *z*, and the scattered light is collected in the GNR *zy* plane. After averaging over the scattered polarizations, the Raman intensities are given as $I\propto 0.5\frac{{n}_{j}+1}{{\omega }_{j}}(| {{\bf{R}}}_{{\rm{zz}},j}({E}_{{\rm{exc}}}){| }^{2}+| {{\bf{R}}}_{{\rm{zy}},j}({E}_{{\rm{exc}}}){| }^{2})$. For the plots of Raman spectra, Lorentzian broadening of the peaks has been applied.

### Device fabrication

The devices were prepared on highly doped single-side polished Si substrates with 300-nm-thick thermally grown SiO_2_. First, 150 × 150 μm^2^-sized contact pads were patterned using optical lithography. For the contact pads, we used 10 nm of titanium for adhesion followed by 50 nm of gold. Subsequently, the aligned GNR heterojunction film was transferred with known orientation onto the substrate^25^. The GNR alignment was checked by polarized Raman measurements (Supplementary Note 4). Electron beam lithography was used to define the source and drain electrodes. For this purpose, the samples were coated with a double layer of poly(methyl methacrylate) (PMMA). The bottom layer (molecular weight 250 kg/mol) is more sensitive than the top layer (molecular weight 950 kg/mol), resulting in an undercut that facilitates lift-off processing. The PMMA-coated substrates were exposed in an electron beam writer, developed, and a 10-nm layer of titanium and 30-nm layer of gold were deposited by thermal evaporation. Subsequently, the metal was removed from the unexposed regions of the sample using a lift-off process.

### UHV transport characterization

Prior to the UHV transport characterization, the SiO_2_/Si wafers with GNR heterojunction devices were glued (silver epoxy) onto sapphire plates, which were mounted onto omicron-type sample holders. The sample holders were equipped with five spring-contact pins mounted at one end (see Fig. 5a). The source and drain contact pads on the sample were each connected by 25 μm Au wire with one pin of the spring contact. Similarly, the back-gate contact was attached to one pin. Upon insertion into the UHV cryostat sample receptacle, the five pins make contact with BNC-type feedthroughs that connect the device inside the UHV chamber to the electronics outside. For each device, we only used three pins (source, drain, and gate). For the application of *V*_g_ and the measurement of the gate leakage current, a Keithley 2400 source measure unit (SMU) was employed. Another SMU of the same type was used for the application of *V*_d_ and the measurement of *I*_d_. Field-effect mobility was determined as: *μ*_DTM_ = *g*_m_(*L*/*W*)(1/*C*_g_*V*_d_), where *C*_g_ is the capacitance per unit area of the SiO_2_ back-gate dielectric and *g*_m_ = ∂*I*_d_/∂*V*_g_ is the transconductance.

### Schottky and tunneling barrier heights

In our FETs, charge carriers are injected from the metal source contact to either quasi-metallic (14-AGNRs, 21-AGNRs, ...) or semiconducting 7-AGNR segments and then transported through a sequence of 7-AGNRs tunneling barriers. Below we compare the Schottky barrier heights of the 14-AGNR/metal and the 7-AGNR/metal contacts with the tunneling barrier height Φ_b_. The estimation of the Schottky barriers for carrier injection from the contacts to GNRs and the tunneling barrier Φ_b_ requires the information of the transport bandgap and the valence and conduction band offsets (VB_1_ and CB_1_, respectively). The transport bandgap strongly depends on the substrate via the screening of the Coulomb interaction^51,52^. As a consequence, the bandgap can range from ~2.3 to 2.5 eV for 7-AGNRs on Au(111) substrate to ~3.7–3.9 eV for isolated 7-AGNRs^14,51–54^. The bandgap of 14-AGNRs on Au is ~0.2 eV and for an isolated 14-AGNR it is around 0.7 eV^14^. For 7-AGNRs on SiO_2_, theory predicts that the bandgap is ~3.3 eV, that is about 85% smaller than for the isolated 7-AGNR^52^. If we scale down the bandgap for isolated 14-AGNRs by 85%, we obtain 0.6 eV, which is valid for 14-AGNRs on SiO_2_. For estimation of the tunneling barrier Φ_b_ on SiO_2_, we assume electron–hole symmetry with the chemical potential of 7-AGNRs and 14-AGNRs lying symmetrically in the gap. This estimation yields Φ_b_ = 1.35 eV as a tunneling barrier for both electrons and holes and ignores the effects of charged impurities in the SiO_2_ that may affect the GNR bandgap and the position of *E*_F_^55^.

We assume that the metal contacts in our FETs provide the same screening as the Au substrate used for GNR synthesis and result in the same bandgaps and energy offsets for the VB_1_ and CB_1_. The VB_1_ and CB_1_ in turn provide the Schottky barriers for hole and electron injection, respectively. The GNR bandgaps at the contact region are just ${E}_{{\rm{g}}}^{7\mbox{-}{\rm{AGNR}}}=2.4$ eV and ${E}_{{\rm{g}}}^{14\mbox{-}{\rm{AGNR}}}=0.2$ eV for 7-AGNRs and 14-AGNRs, respectively, as derived from STS measurements^12,14,53^. It is thus clear that the Schottky barrier for the 14-AGNR/metal interface is much smaller compared to the Φ_b_ and therefore has negligible effect in our devices. For 7-AGNR/metal interface from the ARPES spectra, we obtain VB${\,}_{1}^{7\mbox{-}{\rm{AGNR}}}=0.8$ eV^37^, and since CB${\,}_{1}^{7\mbox{-}{\rm{AGNR}}}={E}_{{\rm{g}}}^{7\mbox{-}{\rm{AGNR}}}-$VB${\,}_{1}^{7\mbox{-}{\rm{AGNR}}}$, we have CB${\,}_{1}^{7\mbox{-}{\rm{AGNR}}}=1.6$ eV. Therefore, the Schottky barriers for 7-AGNR/metal for electron and hole injection are 1.6 and 0.8 eV, respectively. The Schottky barrier for the 7-AGNR to metal contacts is comparable to Φ_b_. Considering that we have many more heterojunctions than Schottky barriers between source and drain, GNR heterojunctions dominate device resistance.

### Model for tunneling transport

To calculate the tunneling probability *P*(*E*) through a trapezoidal barrier, we used the following expression^36^:

4 $$P(E) 	=A\exp \left(-\frac{2}{\hbar }\mathop{\int}\nolimits_{0}^{d}\sqrt{2m[\varphi (x,V)-E]}{\rm{d}}x\right)\\ 	=A\exp \left(-\frac{4d\sqrt{2m}}{3\hbar eV}\left[{({{{\Phi }}}_{{\rm{b}}}-E)}^{3/2}-{({{{\Phi }}}_{{\rm{b}}}-E-eV)}^{3/2}\right]\right).$$

This equation is valid only for the case when the tunneling occurs between the edges of the barrier, i.e., the tunneling length is equal to the geometrical length of the barrier *d*. This is the case if Φ_b_ − *E* > 0 and Φ_b_ − *E* − *eV* > 0. Depending on the sign of *V*_d_, the barrier can either decrease or increase by the amount ∣*eV*∣ along the tunneling path. Since the situation is symmetrical with respect to the direction of the applied voltage, we will consider in the following the case of *V* ≥ 0. In the case Φ_b_ − *E* > 0 and Φ_b_ − *E* − *eV* < 0, the carrier has to tunnel under the triangular barrier and the transmission coefficient is described by the Fowler–Nordheim theory^56^. In Eq. (4), *d* indicates the barrier length, *m* is the charge carrier effective mass inside the barrier, *φ*(*x*,*V*) = Φ_b_ + (*x*/*d*) ⋅ (−*eV*) is the barrier height at coordinate *x*, *V* is the applied voltage, and Φ_b_ denotes the barrier height at *x* = 0. In the case of a triangular barrier, the integration over *x* in the exponent of Eq. (3) should be performed from zero till the value *x*_*c*_ determined by the condition *φ*(*x*_*c*_, *V*) − *E* = 0. The expression for *P*(*E*) then reads,

5 $$P(E)=A\exp \left(-\frac{4d\sqrt{2m}}{3\hbar eV}{({{{\Phi }}}_{{\rm{b}}}-E)}^{3/2}\right),$$

in agreement with the exponent in Eq. (4) of ref. ^32^. The integral over *E* in Eq. (1) is to be calculated in the range *E* ≥ 0, *E* ≥ −*eV*. This integration was performed numerically. The position of the Fermi level *E*_F_ in Eq. (1) is determined by the carrier concentration in the channel, which in turn is controlled by *V*_g_. The carrier concentration *n* in the channel of the heterojunction FET is given by *n* = *n*_0_ + *V*_g_*C*_g_/*e*. Approximating the density of states by a constant *ρ*, we have $n-{n}_{0}=\rho ({E}_{{\rm{F}}}-{E}_{{\rm{F}}}^{0})$ where *n* − *n*_0_ is the change in the carrier concentration induced by the gate voltage and ${E}_{{\rm{F}}}-{E}_{{\rm{F}}}^{0}$ is the Fermi level shift induced by the gate voltage. Rearranging the above equation yields ${E}_{{\rm{F}}}={E}_{{\rm{F}}}^{0}+(n-{n}_{{\rm{0}}})/\rho$. Substituting *n* = *n*_0_ + *V*_g_*C*_g_, we get ${E}_{{\rm{F}}}={E}_{{\rm{F}}}^{0}+{C}_{{\rm{g}}}{V}_{{\rm{g}}}/(e\rho )$. We set *α* = *C*_g_/(*eρ*) and take into account that the source–drain voltage also affects the gate potential at a given position along the channel. For instance, a barrier close to the source contact experiences a different potential than a barrier close to the drain contact. Thus, the gate voltage dependence is modeled in our fit as ${E}_{{\rm{F}}}({V}_{{\rm{g}}},{V}_{{\rm{d}}})={E}_{{\rm{F}}}^{0}(T)+\alpha (T)({V}_{{\rm{g}}}+\beta {V}_{{\rm{d}}})$. This relation assumes that the *E*_F_ is a linear function of carrier concentration, which is always true for a sufficiently small range of *E*_F_, e.g., for a small gate voltage range. In all our fits of *I*_d_ – *V*_d_ curves, the variation of ${E}_{{\rm{F}}}^{0}$ was within 100 meV relative to band edge, i.e., small as compared to Φ_b_. The product *αβ* accounts for the asymmetry between the source and drain contacts, with the former taken as the reference potential against which both gate potential *V*_g_ and drain potential *V*_d_ are applied.

### Sub-threshold swing and sensitivity

The sub-threshold swing $S=\partial {V}_{{\rm{g}}}/\partial {\mathrm{log}}_{10}({I}_{{\rm{d}}})=\partial {V}_{{\rm{g}}}/\partial {\psi }_{{\rm{S}}}\times \partial {\psi }_{{\rm{S}}}/\partial {\mathrm{log}}_{10}({I}_{{\rm{d}}})$, where *V*_g_ is the gate voltage, *I*_d_ is the drain current, and *ψ*_S_ is the surface potential. A back-gated FET structure with channel length comparable to oxide thickness leads to poor gating efficiency: ∂*ψ*_S_/∂*V*_g_ ≪ 1. We report therefore an analysis of the channel referenced sub-threshold swing: ${S}_{{\rm{ref}}}=e^{-1}\partial {E}_{{\rm{F}}}/\partial {\mathrm{log}}_{10}({I}_{{\rm{d}}})$. For the *L* = 200 nm device at room temperature, change of *V*_g_ by 70 V leads to the modulation of *I*_d_ from 33 to 265 nA (Fig. 3d). Such *V*_g_ change in turn corresponds to the charge carrier density Δ*n* = 5 × 10^12^ cm^−2^ or 0.033 electrons per 14-AGNR unit cell, resulting in the Fermi level shift by ~27 meV from the band edge. Therefore, the modulation of *I*_d_ by one decade corresponds to a 34-meV shift of the Fermi level, that is *S*_ref_ = 34 meV/dec.

The sensitivity *s* is defined as the change in output per unit of adsorbate. For our experiments, it is appropriate to take *s* = ∂*I*_d_/∂*q*, where *q* is adsorbate density. Due to the nonlinear nature of charge transport in GNR heterojunction arrays, *s* is not a constant. We take a representative value for the first Li exposure of an *L* = 200 nm GNR array (Fig. 5g). A single Li dose of 0.1 Å corresponds to a 70 V shift in graphene neutrality point (Supplementary Fig. 12), corresponding to a charge carrier density Δ*n* = 5 × 10^12^ cm^−2^. The GNR current change at *V*_d_ = 6 V and *V*_g_ = 70 V is Δ*I*_d_ = 12 nA (Fig. 5g). Assuming that Li adatoms donate 1 electron, the sensitivity is thus *s* = 2.4 × 10^−12^ nA cm^2^. The resolution *r* = Δ*i*_d_/*s* is defined as the smallest change in adsorbate that can be measured and requires knowledge of both sensitivity *s* and current noise Δ*i*_d_. In our experiments, we find a current noise of approximately Δ*i*_d_ = 2 pA, yielding a resolution of *r* = 8 × 10^8^ cm^−2^.

## Supplementary information

Supplementary information

## Data Availability

The datasets generated during and/or analyzed during the current study are available from the corresponding author on reasonable request.
