# Supplementary material for: Tunneling current modulation in atomically precise graphene nanoribbon heterojunctions
Source: Nat Commun. 2021 May 5;12:2542. doi: 10.1038/s41467-021-22774-0 (PMC8099867; doi:10.1038/s41467-021-22774-0)
Supplement: Supplementary file 1 — Supplementary information [file 41467_2021_22774_MOESM1_ESM.pdf]

Supplementary Information: Tunneling current modulation in atomically precise graphene nanoribbon heterojunctions

Boris V. Senkovskiy, Alexey V. Nenashev, Seyed K. Alavi, Yannic Falke, Martin Hell, Pantelis Bampoulis, Dmitry V. Rybkovskiy, Dmitry Yu. Usachov, Alexander V. Fedorov, Alexander I. Chernov, Florian Gebhard, Klaus Meerholz, Dirk Hertel, Masashi Arita, Taichi Okuda, Kenya Shimada, Felix R. Fischer, Thomas Michely, Sergei D. Baranovskii, Klas Lindfors, Thomas Szkopek, Alexander Grüneis

Supplementary Note 1. STM studies of GNR heterojunctions

Supplementary Figures 1a-d display STM images of various atomically-precise aligned GNR heterojunctions on Au(788) where fused quasi-metallic GNRs (14-AGNR and 21-AGNR) are connected by wide-bandgap 7-AGNR segments. The spatial density of GNR heterojunctions varies slightly depending on the sample area (Fig. 2a of the main text and Supplementary Figure 1e). The distribution of the 7-AGNR tunneling barrier lengths in the STM images is shown in the Supplementary Figure 1f (summarized result from Fig. 2a of the main text and Supplementary Figure 1e). The average barrier length is approximately 4 nm.

Supplementary Figures 2a,b show the STM image of aligned GNR heterojunctions and the tunnelling conductance dI/dV spectra (numerically differentiated from $I(V)$ curves) acquired at selected locations of the STM image. Each spectrum is the result of averaging over 25 point spec-

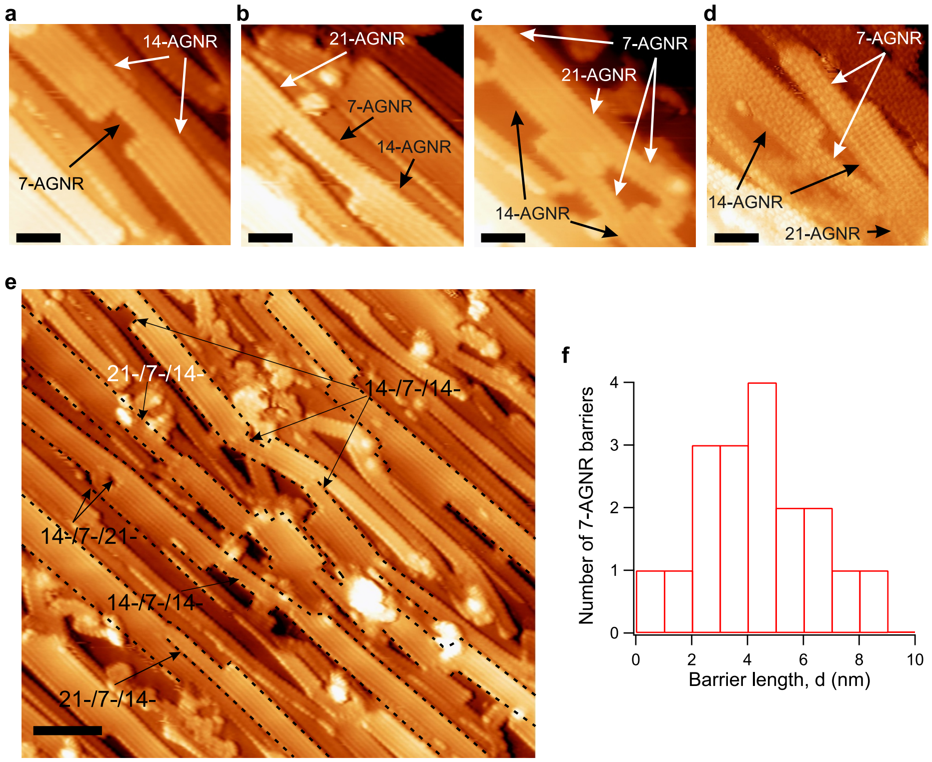

Supplementary Figure 1 STM studies of GNR heterojunctions on Au(788). a-c STM images at sample bias V_s = -0.8 V, tunneling current I_t = 1 nA. **d** STM image at V_s = -1 V, I_t = 1 nA. The scale bar is 2 nm. **e** STM image of the area with a high density of GNR heterojunctions. Possible paths for charge carriers are showed by the dashed black lines. The scale bar is 5 nm. V_s = -0.8 V, I_t = 1 nA. **f** Statistical analysis of the length of 7-AGNR barriers d from STM images in **e** and in Fig. 2a of the main text.

tra at each location. Note, the dI/dV spectra contain the contribution from Au states. Moreover, the fine features of dI/dV curves in our room temperature data is smoothed in comparison to low-temperature STS studies. [1,2] Nevertheless, one can see a distinct behavior of differential conductance dI/dV curves, reflecting the local density of states (LDOS), for insulating 7-AGNR. Their common feature is their much lower differential conductance around $+/-$ 0.5 eV around Fermi-level compared to the wider quasi-metallic AGNR segments. The 7-AGNRs display a shoulder at V_{s} about -0.6 V which is likely to indicate the valence band maximum (in agreement with the low-temperature STS measurements [1]). Supplementary Figures 2c,d depict the dI/dV maps (reflecting the spatial distribution of LDOS) at sample bias $V_{\mathrm{s}} = -/+$ 0.5 V and illustrate that all 7-AGNR segments are gapped (visible as dark colour) irrespective of their configuration. Apparently, there is no strong effect of the configuration of GNR heterojunctions on the energy band off-sets and the tunneling barrier height. Supplementary Figure 2d illustrates electronic states (shown by black stars) at the short termini (zig-zag edges) of the 7/14-AGNR junctions originating from 14-AGNRs. [2,3]

Supplementary Note 2. ARPES studies of GNR heterojunctions

Our initial system is 7-AGNRs on Au(788), which we have studied by ARPES in a previous work. [4] To visualize the GNR electronic band structure by ARPES one must take into account the photoemission matrix element, which is strongly dependent upon $\mathbf{k}_{\perp}$, the in-plane momentum perpendicular to the GNR axis. [4] The matrix element dependence is such that each 1D sub-band of the GNRs is visible in a narrow range of $\mathbf{k}_{\perp}$. The ARPES spectrum of 7-AGNRs on Au(788)

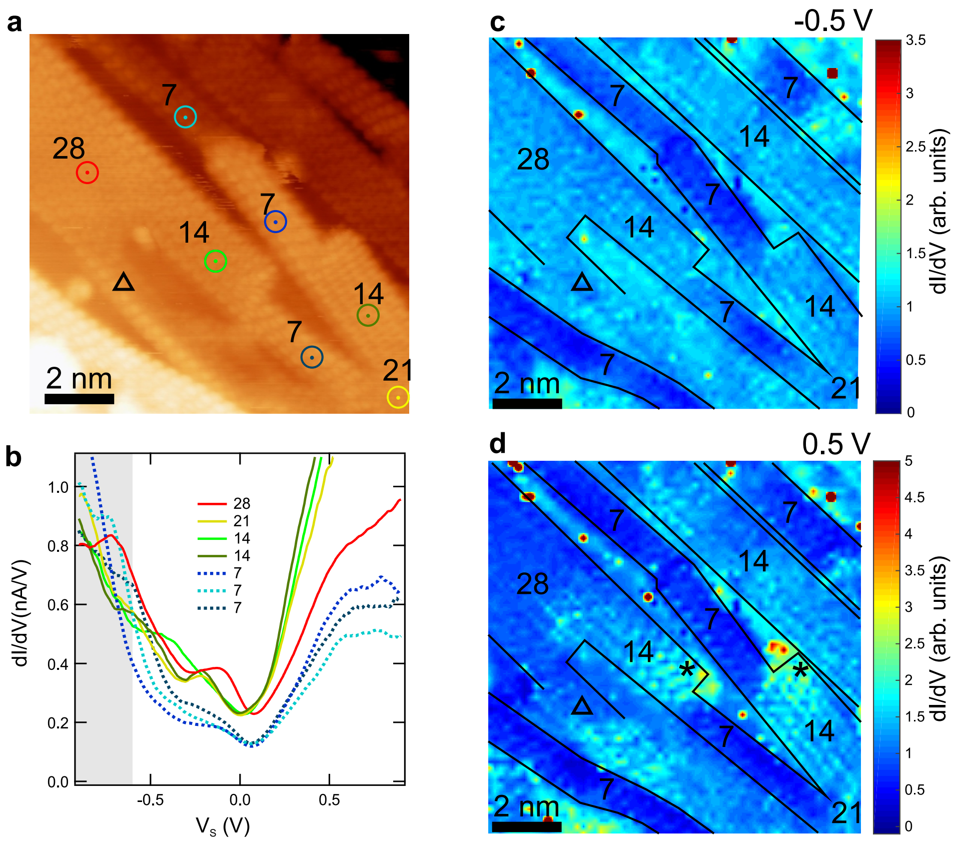

Supplementary Figure 2 STS studies of aligned GNR heterojunctions on Au(788). **a** STM image at sample bias $V_s = -1$ V, tunneling current $I_t = 1$ nA. **b** Numerically differentiated dI/dV spectra (each spectrum is the average of 25 single point spectra) acquired at different locations marked in panel **a**. The measurements were done at room temperature. The labels 7, 14, 21, and 28 refer to 7-, 14-, 21- and 28-AGNRs, respectively. The dashed curves come from 7-AGNR segments and the solid curves from quasi-metallic GNRs. The gray background in panel **b** indicates $V_s < -0.6$ V, where the 7-AGNR valence band edge starts contribute in LDOS. **c, d** Color-coded dI/dV maps obtained at **c** $V_s = -0.5$ V and **d** at $V_s = 0.5$ V at room temperature ($I_t = 0.5$ nA). 7-AGNR segments have low intensity (if they are not lying exactly on the Au step edges, as 7-AGNR shown by the black triangle). Black stars in panel **d** denote the 14-AGNR states having larger intensity at the zigzag edges. Black triangle denotes a 7-AGNR lying on the step edge of the Au(788) crystal, which therefore appears bright on dI/dV maps.

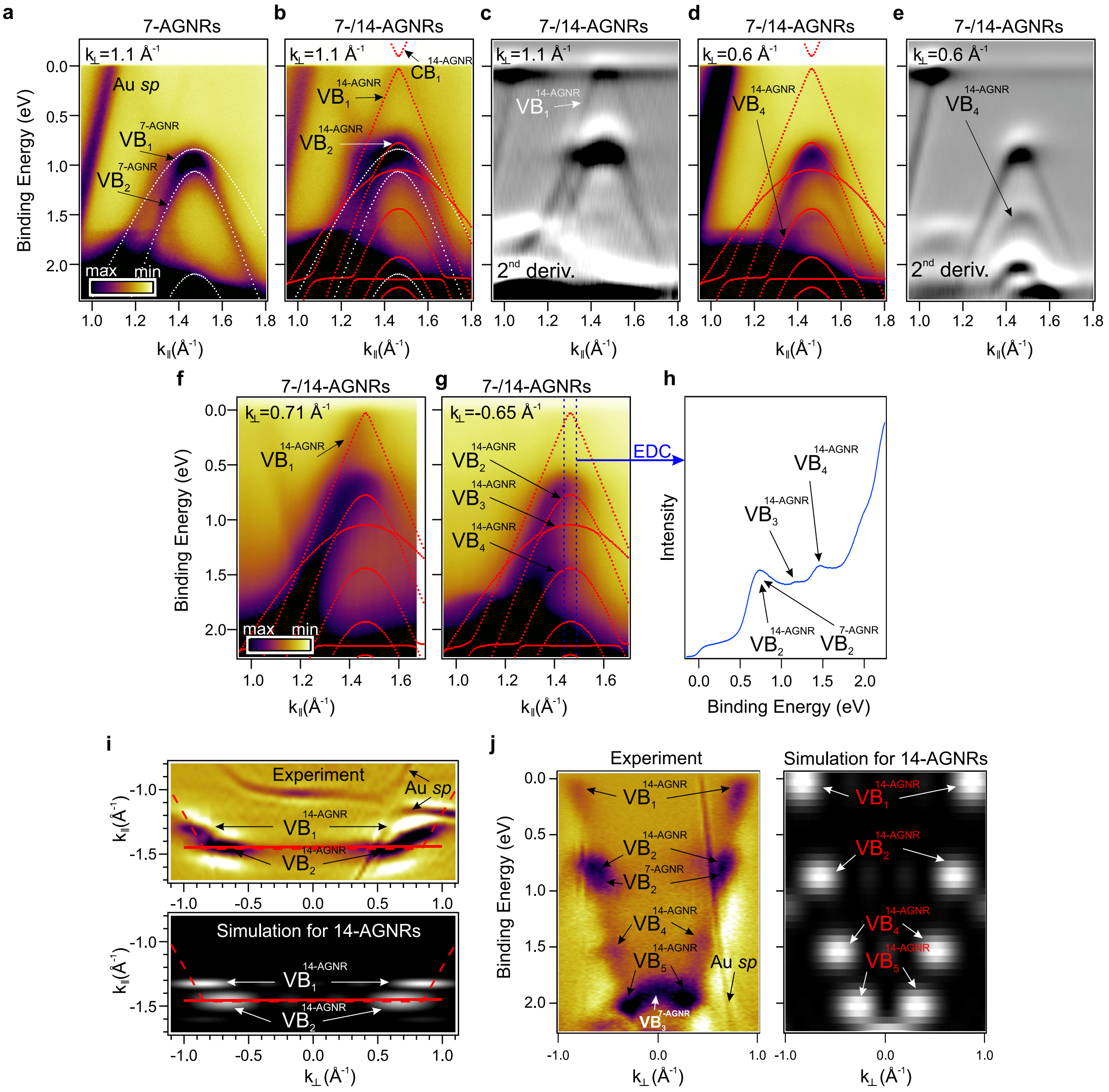

Supplementary Figure 3 ARPES studies of GNR heterojunctions on Au(788). a ARPES scan of the 7-AGNRs on Au(788) initial system measured at $\mathbf{k}_\perp = 1.1$ Å^{-1} along with calculations (white dashed lines). $VB_1^{7\text{-AGNR}}$ and $VB_2^{7\text{-AGNR}}$ indicate the first and second valence sub-band of 7-AGNRs, respectively. **b** ARPES scan of the same sample after *in situ* fusion. $VB_1^{14\text{-AGNR}}$ and $VB_2^{14\text{-AGNR}}$ indicate the first and second valence sub-bands of 14-AGNRs in the calculations (red dashed lines), respectively. **c** Second derivative by energy of the data in **b**. **d** ARPES scan of the same sample but at $\mathbf{k}_\perp = 0.6$ Å^{-1}. **e** Second derivative by energy of the data in **d**. **f, g** ARPES scans of the fused GNRs on Au(788) (see Methods) at **f** $\mathbf{k}_\perp = 0.71$ Å^{-1} and **g** at $\mathbf{k}_\perp = -0.65$ Å^{-1}. **h** EDC of the scan in **g** around the center of the second BZ of GNRs. The width of the spectra taken in EDC is shown by the blue dashed lines. **i** Experimental ARPES map at the binding energy of 0.8 eV representing the second derivatives (top) and the corresponding cut of the simulated map for 14-AGNRs (bottom). The intensity from $VB_1^{14\text{-AGNR}}$ and $VB_2^{14\text{-AGNR}}$ is indicated. The red dashed line depicts the BZ border of graphene. **j** Experimental cut of the ARPES map along the thick red line shown in **i**, and the corresponding cut of the simulated map for 14-AGNRs ($\mathbf{k}_\parallel = -1.47$ Å^{-1}). The sub-bands of 14-AGNRs and 7-AGNRs that should be visible in the cut are denoted.

in Supplementary Figure 3a was accumulated along the GNR axis ($\mathbf{k}_{||}$ in the momentum space) at $\mathbf{k}_{\perp} \sim 1$ Å^{-1} to simultaneously visualize the two topmost (closest to the Fermi level, E_{F}) valence sub-bands of 7-AGNRs, labeled as $\mathrm{VB_1}^{\text{7-AGNR}}$ and $\mathrm{VB_2}^{\text{7-AGNR}}$, respectively. The calculated band structure of 7-AGNRs is shown by the white dashed lines. After lateral fusion performed *in situ* we observe a new linearly dispersing band, which touches E_{F} (Supplementary Figure 3b). In Supplementary Figure 3c we show the second derivative of the intensity with respect to energy to enhance the contrast. Fig. 2c in the main text shows the second derivative of the same spectrum with respect to momentum to better see the linear band near the Fermi level. The ARPES scan at $\mathbf{k}_{\perp} \sim 0.6$ Å^{-1} (Figs. S3d,e) reveals another new 1D sub-band with parabolic dispersion which does not originate from 7-AGNRs. [4] Note that this band was also visible in earlier ARPES studies of 7-AGNRs on Au(788) as a result of unintended fusion. [1,5,6] The calculated band structure of 14-AGNRs is shown in Supplementary Figures 3b,d by the red dashed lines. By comparison with DFT calculations, we see that the energies of the new linear and parabolic bands in ARPES spectra fit to the first ($\mathrm{VB_1}^{\text{14-AGNR}}$) and fourth ($\mathrm{VB_4}^{\text{14-AGNR}}$) valence sub-bands of 14-AGNRs, correspondingly. These sub-bands of 14-AGNRs are easy to distinguish since they do not overlap with sub-bands of 7-AGNRs.

Supplementary Figures 3f-j shows ARPES data on the aligned GNR heterojunction sample that was synthesized in the ultra-high vacuum (UHV) Raman apparatus (Supplementary Note 4) and then transferred to the ARPES facility. For this system we measured ARPES spectra at $\mathbf{k}_{\perp} = 0.71$ Å^{-1} (Supplementary Figure 3f) where the linear band $\mathrm{VB_1}^{\text{14-AGNR}}$ is pronounced, and at $\mathbf{k}_{\perp} = -0.65$ Å^{-1} (Supplementary Figure 3g), where the 14-AGNR valence sub-bands with larger binding

energies are visible. In the energy distribution curve (EDC) taken from the scan in Supplementary Figure 3g around the center of the second Brillouin zone (BZ) of AGNRs, we can observe the maxima of 14-AGNR valence sub-bands (Supplementary Figure 3h). Their energy positions fit to the $VB_3^{14\text{-AGNR}}$ and $VB_4^{14\text{-AGNR}}$.

To unambiguously attribute the newly appearing sub-bands to 14-AGNRs, we measured ARPES maps and performed the simulations of the photoemission intensity, as we previously did for pristine and boron-doped 7-AGNRs on Au(788). [4,7] Supplementary Figure 3i depicts the experimental ARPES map of fused GNRs on Au(788) (top) and the ARPES intensity simulation for 14-AGNRs (bottom) at a binding energy of 0.8 eV. Experiment and simulation are consistent in the momentum space position of several spectral features of 14-AGNRs, namely the sub-bands $VB_1^{14\text{-AGNR}}$ and $VB_2^{14\text{-AGNR}}$. Taking the energy cut of the ARPES map along $\mathbf{k}_\perp$ at the middle of second BZ of GNRs ($\mathbf{k}_\parallel = -1.47$ Å^{-1}), as shown by the thick red line in Supplementary Figure 3i, we can visualize the quantization of photoemission intensity of 14-AGNRs sub-bands, which is in a good agreement with simulations. In this cut two sub-bands of 7-AGNRs ($VB_2^{7\text{-AGNR}}$ and $VB_3^{7\text{-AGNR}}$) are also visible.

Supplementary Note 3. ARPES studies of Li doped GNR heterojunctions

Supplementary Figure 4 displays the ARPES measurements of Li doped fused GNRs on Au(788). The Li doping was performed with the sample presented in Supplementary Figures 3f-i. Due to the variation of photoemission intensity with momentum and energy, we have to select a certain $\mathbf{k}_\perp$

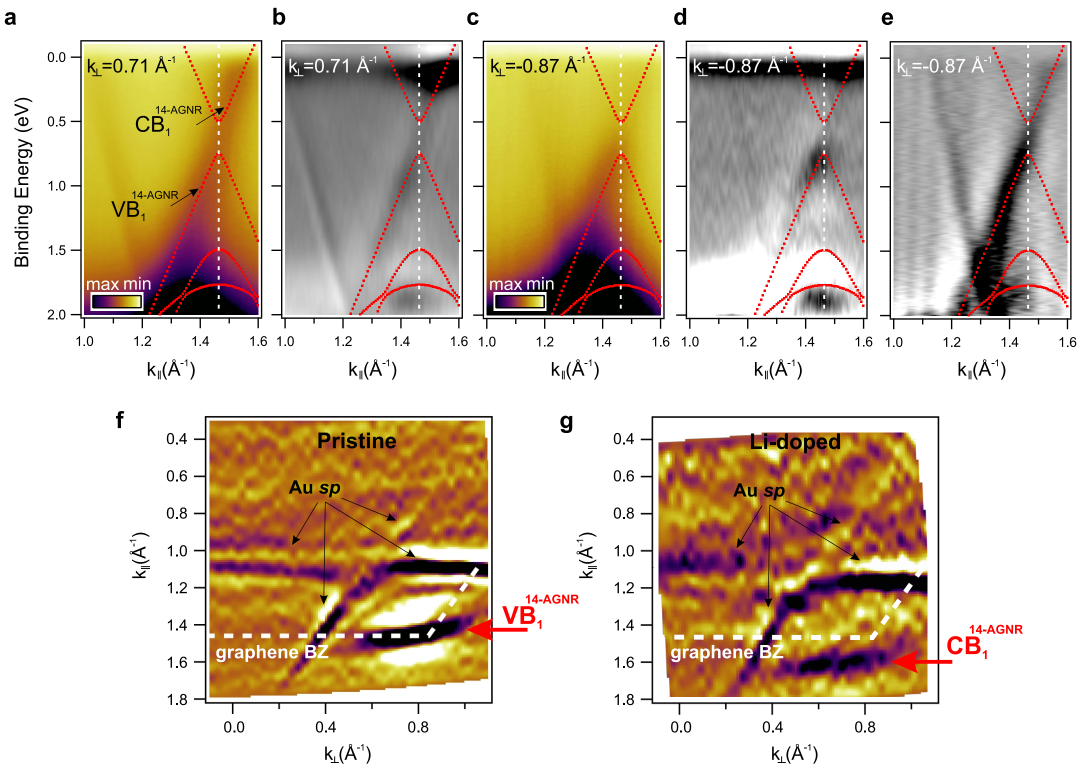

Supplementary Figure 4 ARPES studies of Li doped GNR heterojunctions on Au(788). **a, b** ARPES scans at $\mathbf{k}_\perp = 0.71$ Å^{-1}. **c-e** ARPES scans at $\mathbf{k}_\perp = -0.87$ Å^{-1}. Second derivatives with respect to energy are shown in **b, d** and with respect to momentum in **e**. The calculated band structure of 14-AGNRs (the red dashed lines) is overlaid on the ARPES spectra. The calculated bands were shifted in energy to match the experimental intensity. The vertical white dashed line denote the middle of the second GNR BZ (which corresponds to the BZ edge of graphene). **f, g** ARPES maps of fused GNRs on Au(788) taken at the Fermi level for **f** pristine and **g** Li-doped samples. The intensity of $VB_1^{14\text{-AGNR}}$ and $CB_1^{14\text{-AGNR}}$ is indicated by the red arrows. The BZ of graphene and the *sp*-states of Au substrate are indicated for reference. ARPES maps are composed from the second derivatives by energy to enhance the spectral features.

to visualize both valence and conduction band edges of 14-AGNRs ($VB_1{}^{\text{14-AGNR}}$ and $CB_1{}^{\text{14-AGNR}}$, correspondingly) after Li doping ($\sim$ 1 Å). This condition is fulfilled at $\mathbf{k}_\perp \sim 0.7$ Å^{-1}, as shown in Supplementary Figure 4a. The second derivative with respect to energy of this spectrum is shown in Supplementary Figure 4b, and the second derivative with respect to momentum is shown in Fig. 5c (right) of the main text. Supplementary Figures 4c-e depicts the ARPES spectrum and its second derivatives of the same sample but at $\mathbf{k}_\perp = -0.87$ Å^{-1}, where the intensity of the $CB_1{}^{\text{14-AGNR}}$ vanishes and only the $VB_1{}^{\text{14-AGNR}}$ is clearly visible. The calculated $VB_1{}^{\text{14-AGNR}}$ in Supplementary Figure 4 is shifted by 0.7 eV. Due to the matrix element effect, we observe photoemission intensity only for the 'left' branch of $VB_1{}^{\text{14-AGNR}}$ and the 'right' branch of $CB_1{}^{\text{14-AGNR}}$, relative to the middle of the second BZ of GNR (white vertical dashed line in Supplementary Figures 4a-e). Here, 'left' and 'right' branches refer to $\mathbf{k}_\parallel$ smaller or larger than 1.47 Å^{-1}, respectively. The fact that the $VB_1{}^{\text{14-AGNR}}$ and $CB_1{}^{\text{14-AGNR}}$ branches are visualized for pristine and Li-doped samples, correspondingly, can be inferred from the ARPES maps taken at the Fermi level (Supplementary Figures 4f,g). It can be seen that the $CB_1{}^{\text{14-AGNR}}$ in the Li-doped sample appears at larger $\mathbf{k}_\perp$ as compared to the $VB_1{}^{\text{14-AGNR}}$ of the pristine sample. From the ARPES measurement of a Li doped sample we estimate the position of the $CB_1^{14-\text{AGNR}}$ edge as $\sim$ 0.5 eV below the Fermi level, which corresponds to 0.17 electrons per 14-AGNR unit cell.

Supplementary Note 4. Raman studies of GNR heterojunctions

We find that we can observe both, 7- and 14-AGNR Raman modes, if we excite them with a laser of 1.96 eV energy (633 nm), which is slightly below the optical bandgap of 7-AGNRs on Au(788)

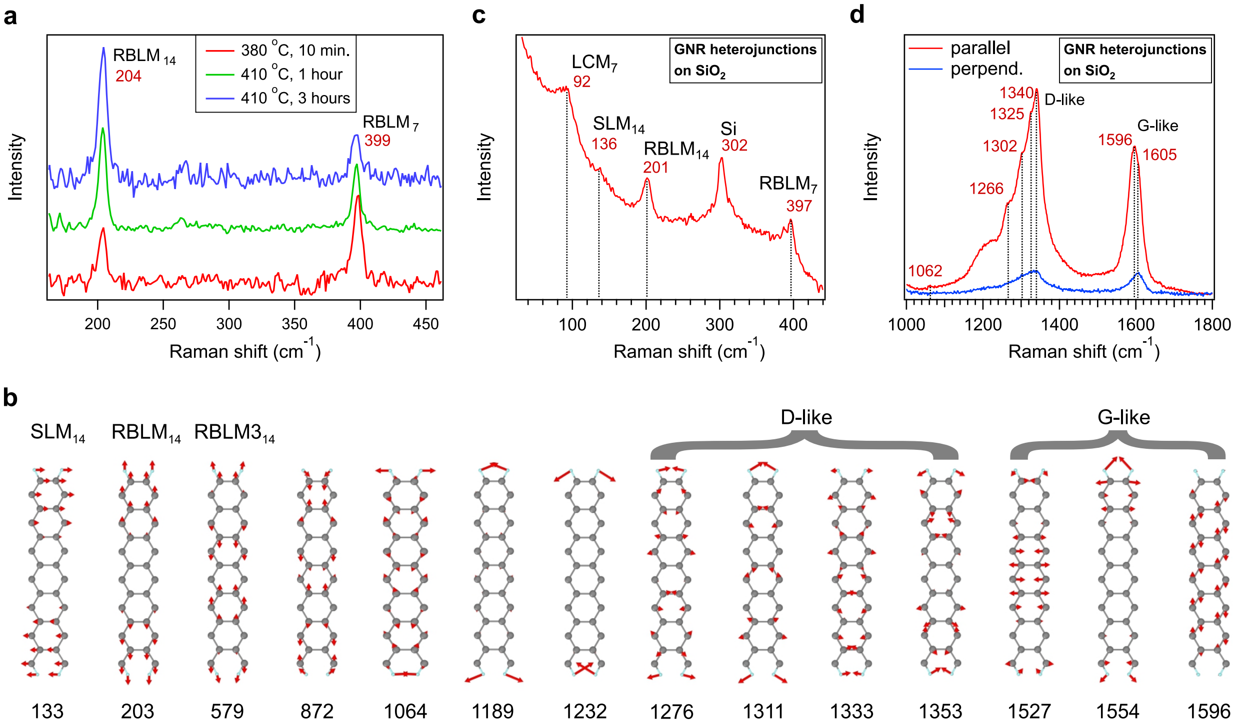

Supplementary Figure 5 Raman studies of GNR heterojunctions. **a** UHV Raman spectra of aligned 7-AGNRs on Au(788) before (red spectrum) and during the lateral fusion indicate increase of the $RBLM_{14}$ intensity and decrease of the $RBLM_7$ intensity at 410 °C. The laser polarization is along the alignment direction of GNR heterojunctions. **b** Eigenvectors of the most intense Raman active modes of 14-AGNR and their respective frequencies (values in cm^{-1}). Red vectors indicate the atomic displacements. **c, d** Raman spectra of aligned GNR heterojunctions on SiO_2/Si (633 nm laser) **c** in the low wavenumber region with the laser polarization parallel to the source-drain direction and **d** in the region of the G- and D-like modes with the laser polarization parallel (red) and perpendicular (blue) to the source-drain direction.

($\sim$ 2.1 eV). [8,9] Supplementary Figure 5a depicts the evolution of RBLM Raman modes of 7- and 14-AGNRs ($RBLM_7$ at 399 cm^{-1} and $RBLM_{14}$ at 204 cm^{-1}, correspondingly) upon the lateral fusion of 7-AGNRs/Au(788) at 410 °C at different fusion times. Initial system was synthesised at 380 °C for 10 minutes and its Raman spectrum consists primarily of 7-AGNR derived vibrational modes (red spectrum in Fig. 2d of the main text). The presence of the $RBLM_{14}$ peak near the $RBLM_7$ (Fig. 2d of the main text and Supplementary Figure 5a) is evidence of unintended lateral fusion already occurring during the synthesis of 7-AGNRs on Au(788). We found that a relatively low temperature is sufficient to perform conversion of a substantial amount of 7-AGNRs into wider AGNRs, as can be seen from the Raman spectra in Supplementary Figure 5a, demonstrating a strong decrease of the $RBLM_7$ intensity and simultaneously a strong increase of the $RBLM_{14}$ intensity. After $\sim$ 3 hours at 410 °C the $RBLM_{14}$ peak reaches the maximum intensity, which is $\sim$ 2.4 times larger compared to the initial system. At the same time, the $RBLM_7$ peak intensity is decreased by $\sim$ 2 times. This means that more than 50 % of 7-AGNRs are fused, and we take this system as final GNR heterojunctions for our FETs. Longer fusion time and higher temperatures lead to the decrease of both $RBLM_7$ and $RBLM_{14}$ intensity, and to the broadening of these modes.

Supplementary Figure 5b shows the atomic displacements and frequencies of the calculated 14-AGNR phonon modes. The calculated phonon frequencies are in very good agreement with the experimentally observed peaks in the low-frequency and the D-like mode regions of the Raman spectra (Fig. 2d of the main text), but are slightly lower for the high-energy G-like modes. The low-frequency shear-like mode (SLM) of 14-AGNRs (SLM_{14}) cannot be observed in our UHV Raman system. However, this mode can be detected *ex situ* when the aligned GNR heterojunction film

is transferred to a SiO_2/Si substrate. Supplementary Figure 5c shows the Raman spectrum measured using a low wavenumber filter. In this spectrum we identify $RBLM_7$, $RBLM_{14}$ and SLM_{14} peaks, as well as the longitudinal compressive mode of 7-AGNRs [10] (LCM_7). These characteristic Raman features corroborate the high structural quality of the transferred GNR heterojunctions. Supplementary Figure 5d depicts *ex situ* polarized Raman measurements of the GNR heterojunctions on SiO_2/Si. In these Raman measurements, the polarization of the optical field was chosen parallel or perpendicular to the source-drain direction. The large anisotropy in the Raman intensity for the two polarizations confirms the alignment of the GNR heterojunctions in our FETs along the source-drain direction.

Supplementary Note 5. GNR heterojunction devices

Supplementary Figure 6a shows the scanning electron microscopy (SEM) images of the GNR heterojunction devices of different channel length L. The room temperature measurements of the L = 200 nm device are shown in Supplementary Figure 6b,c. From analysis of I_d-V_g curves in Supplementary Figure 6c we obtain that for the gate voltage in the range -60 V < V_g < -70 V the mobility μ_DTM ranges from 5.3×10^{-5} cm^2/Vs at V_d = 3 V to 6.1×10^{-4} cm^2/Vs at V_d = 6 V (averaging between V_g up- and down-sweep measurements). If we compare these values with those obtained at 4K (see discussion of Fig. 3b,c in the main text), we find that at fixed V_d = 6 V the μ_DTM differs by only one order of magnitude. Such a small dependence of charge transport on temperature is also observed in other devices, for example, I_d-V_g curves for the $L = 500$ nm device show in Supplementary Figure 6d,e demonstrate similar behaviour. Notably, we find that in

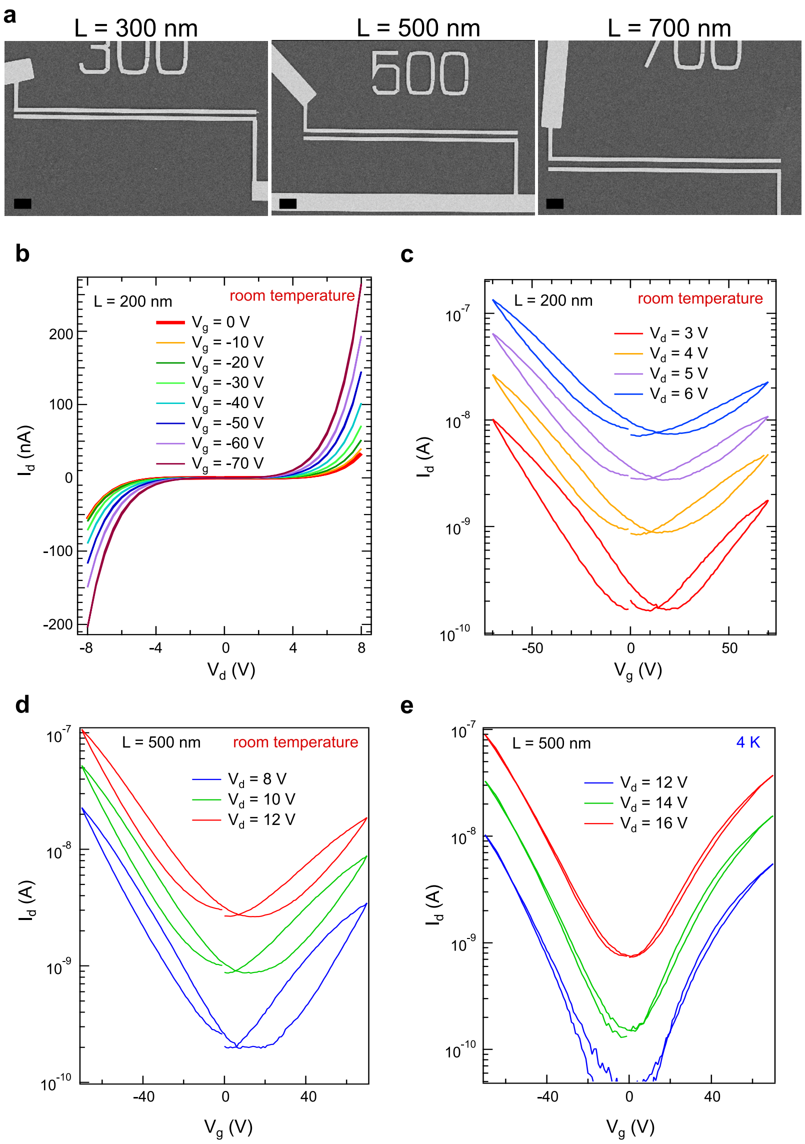

Supplementary Figure 6 GNR heterojunction devices of different channel length. **a** SEM images of the GNR heterojunction devices with $L = 300$, 500 and 700 nm. The scale bar is 2 μm. **b, c** Transport characteristics of the $L = 200$ nm device at room temperature; **b** I_{d}-V_{d} curves at different V_{g}, and **c** I_{d} versus V_{g} at different V_{d}. **d, e** I_{d}-V_{g} characteristics of the $L = 500$ nm device at different V_{d} at **d** room temperature and **e** at 4 K.

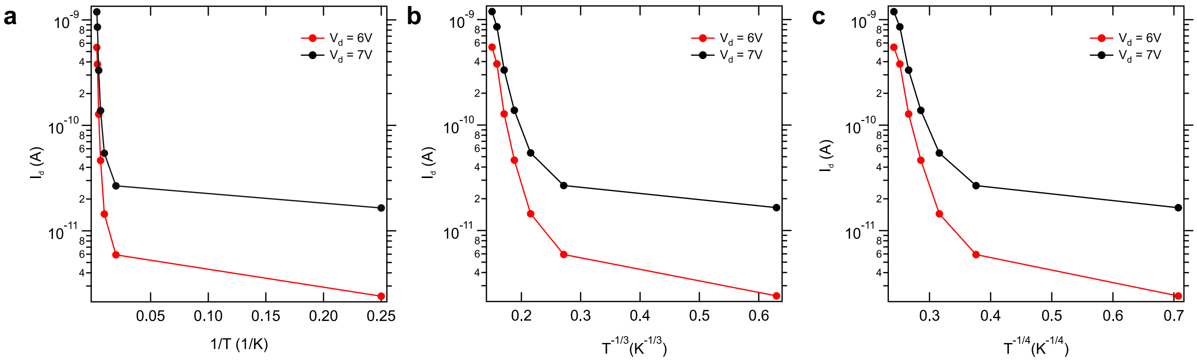

Supplementary Figure 7 Deviation from the Mott hopping transport. Semilogarithmic plots of I_d versus $(1/T)^{(1/N)}$ with **a** N = 1, **b** N = 3 and **c** N = 4 for the L = 350 nm device at V_d = 6 V and 7 V. All curves deviate from a straight line expected for the Mott hopping transport.

our devices, hysteresis with V_g sweep direction disappears at 4 K. This suggests that the origin of the hysteresis at room temperature is the presence of charge-trapping centers at the SiO_2 surface, in agreement with recent studies. [11]

Charge carrier transport in our devices does not agree with Mott variable-range hopping (VRH) model. If the conductance σ is dominated by Mott hopping, it should depend on the temperature T as $\sigma = \sigma_0 \exp(-[T_0/T]^{(1/N)})$, where σ_0 is a constant, T_0 is the characteristic temperature scale and $1/N$ is the hopping index, that depends on the dimensionality. [12] N = 3 and 4 for 2D and 3D systems, respectively. In the case of $N = 1$, Mott's law reduces to an Arrhenius activation law with a well defined activation energy $E_A = k_B T_0$ for hopping, sometimes known as the nearest neighbour hopping (NNH) model. We plot log(I_d) against $(1/T)^{(1/N)}$ with N = 1, 3 and 4 for two different values of V_d (Supplementary Figure 7). Deviation from a straight line indicates that the experimentally observed conduction is not well described by variable range hopping or nearest neighbour hopping models.

To demonstrate the uniformity of our devices we fabricated 3 different wafers with aligned GNR heterojunctions, on which we have measured 14 working devices. Supplementary Figure 8 shows the I_{d}-V_{d} curves of these devices. Supplementary Figure 9 displays the average absolute current $I_{\mathrm{d}}^{\mathrm{avg}}$ at V_{d} = +8 V and V_{d} = −8 V as a function of L for L = 300, 350, 500, and 700 nm across different devices. It is seen that different devices of the same L show almost identical behaviour. The same is true for the L = 100 nm and L = 1000 nm devices (Supplementary Figure 8), that cannot be compared at V_{d} = 8 V. This corroborates the reproducible fabrication of GNR heterojunction devices with uniform quality.

Supplementary Note 6. Comparison with the nuclear tunneling model

The nuclear tunneling model for polaron conduction in organic semiconductors [13] has been recently applied by Richter et al. to explain the charge transport in a network of narrow bandgap 5-AGNRs. [14] This model assumes that the space between overlapping 5-AGNRs is the tunneling barrier. The system in our work has insulating 7-AGNR segments as tunneling barriers between quasi-metallic (14-AGNR and 21-AGNR) segments that have a bandgap, which is even smaller than 5-AGNRs. The question is whether a possible agreement to the model from Richter et al. also implies that conductance in our system is governed by nuclear tunneling (hopping) of polarons between quasi-metallic GNRs. This is not the case because inspection of the fit parameters leads to a contradiction, described in detail below.

Let us try to use Eq. (S1) from Ref. 14 derived for the mechanism of transport induced by

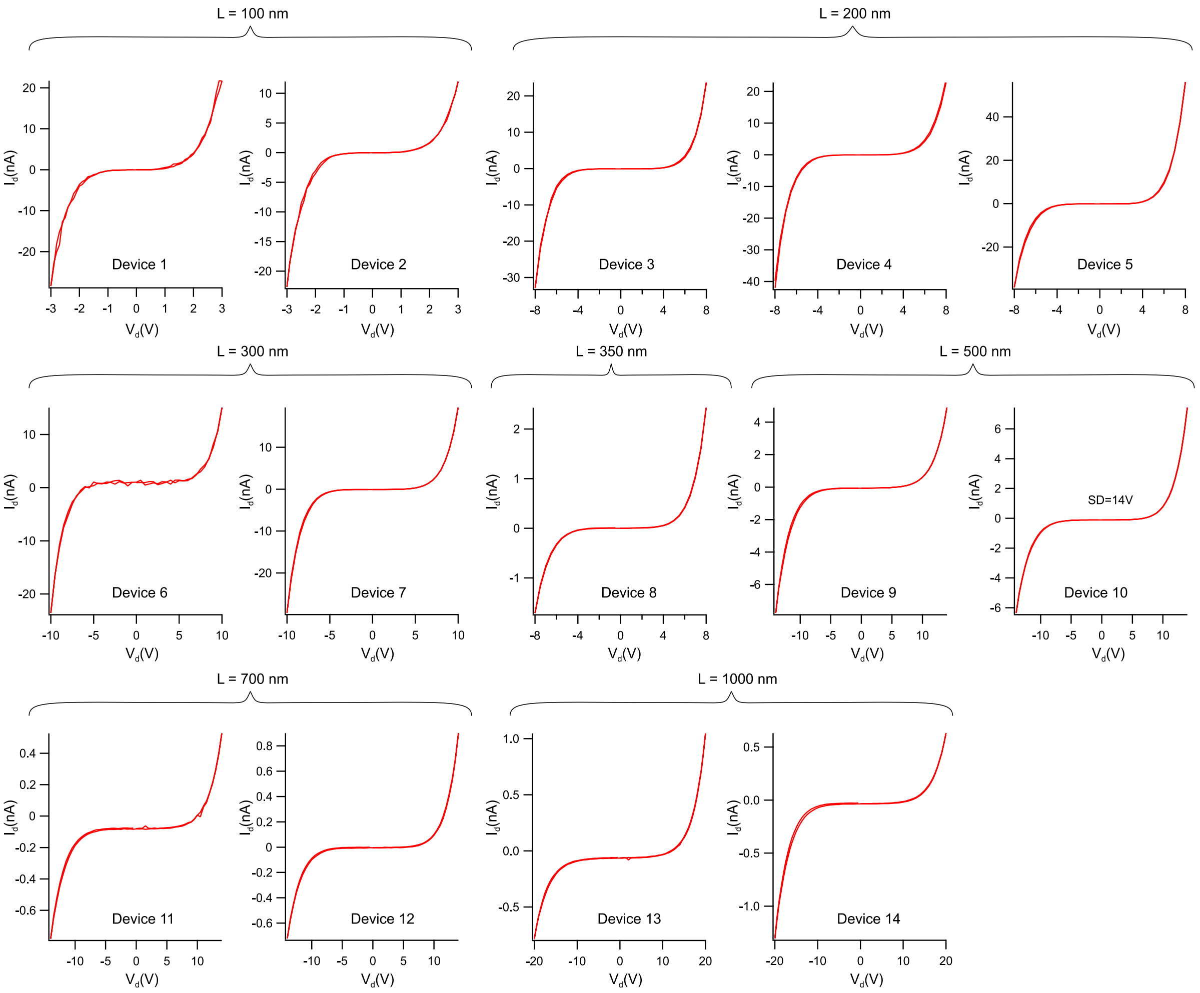

Supplementary Figure 8 Uniform behaviour of GNR heterojunction devices. I_d-V_d characteristics of 14 measured devices of different channel length L at $V_g = 0$ V at room temperature, UHV conditions.

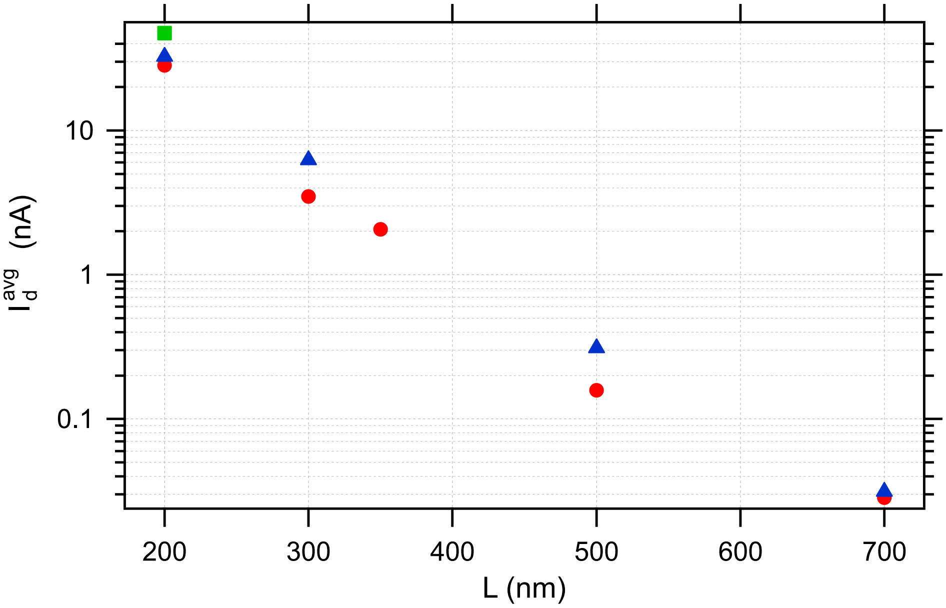

Supplementary Figure 9 Channel length dependence of the drain current. The absolute drain current $I_\mathrm{d}^\mathrm{avg}$ averaged for V_d = 8 V and V_d = −8 V at V_g = 0 V as function of channel length L. Different devices of a given L are indicated by the red, blue and green markers.

nuclear tunneling to fit the temperature dependence of I_d-V_d curves obtained in our study:

$$I_\mathrm{d} = I_0 T^{\alpha+1} \sinh\left(\frac{\gamma e V_\mathrm{d}}{2k_\mathrm{B}T}\right)\left|\Gamma\left(1+\frac{\alpha}{2}+i\frac{\gamma e V_\mathrm{d}}{2\pi k_\mathrm{B}T}\right)\right|^2, \quad \text{(S1)}$$

where γ^{-1} is the number of hopping events between source and drain, parameter α describes the coupling between the charges and the heat bath, and Γ is the complex gamma function. In Supplementary Figure 10a, the fits by Eq. (S1) of our experimental data for the device with the channel length L = 350 nm are shown, and the fit parameters are specified. Furthermore, as has been mentioned by Asadi et al. [13] and by Richter et al., [14] Eq. (S1) leads to a linear dependence of the scaled drain current $I_\mathrm{d}/T^{\alpha+1}$ versus relative energy $eV_\mathrm{d}/k_\mathrm{B}T$ depicted on a log-log scale. In Supplementary Figure 10b, our experimental data in log-log scale agree with this dependence. Nevertheless, it seems not likely that hopping induced by nuclear tunneling is responsible for charge transport in our system, because the parameters necessary for the fits look unrealistic, as explained below. The

agreement depicted in Supplementary Figure 10b also does not necessarily provide evidence for the nuclear-tunneling mechanism, because this log-log behaviour is not unique for this mechanism. Rather, this scaling is truly universal in that other transport models abide by the same scaling rule, for example, thermionic emission in 1-, 2- and 3-dimensional systems (i.e. Richardson equation). The reasonable fits with Eq. (S1) of our experimental data were achieved using the values $\gamma^{-1} = 20$ and $I_0 = 8.5 \times 10^{-37}$ A/K$^{\alpha+1}$ per 25 μm, which is equivalent to $I_0 = 3.2 \times 10^{-32}$ A/K$^{\alpha+1}$m^{-1}. The parameter γ^{-1} represents the number of hopping events in the transport channel between the electrodes (equivalent to M in our tunneling barrier model). The value $\gamma^{-1} = 20$ means that the hopping length (distance between initial and final states in a hop) in our sample should be equal to $350/20 = 17.5$ nm. This length is compatible with the length of fused 14-AGNRs (and to the length of 5-AGNRs from Ref. 14). However, unlike the network of overlapping narrow-bandgap 5-AGNRs measured by Richter et al., [14] our system consists of quasi-metallic GNRs connected by wide-bandgap 7-AGNR segments. Parameter I_0 must capture the dependence of current on the carrier concentration and on the number of conducting channels (related to the device width). In this situation, one expects the value I_0 extracted in our fit to Eq. (S1) to be larger than the value I_0 determined by Richter et al. [14] for 5-AGNR network because the bandgaps of 14-AGNRs and of 21-AGNRs are smaller than the bandgap in 5-AGNRs. [15] As shown by Richter et al., [14] Eq. (S1) fits the data for 5-AGNRs if the value $I_0 = 8.3 \times 10^{-13}$ A/K$^{\alpha+1}$m^{-1} is used. The difference to $I_0 = 3.2 \times 10^{-32}$ A/K$^{\alpha+1}$m^{-1}, which is necessary to describe our data, is about eighteen orders of magnitude in the direction opposite to the expected one. Such a large difference cannot be explained by the fact that the surface coverage by fused quasi-metallic AGNRs in our system

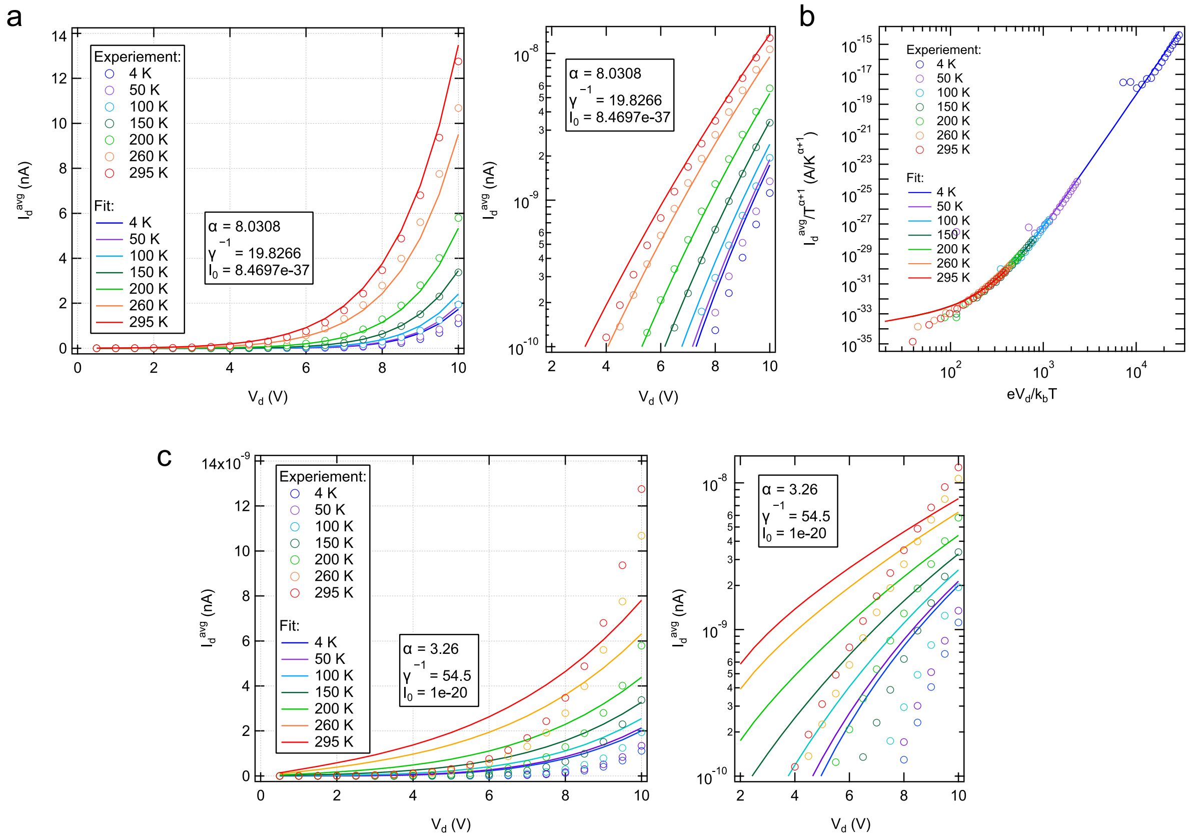

Supplementary Figure 10 Comparison with the nuclear tunneling model. **a** Plot of the average absolute current $I_{\rm d}^{\rm avg}$ versus V_d for the L = 350 nm device at $V_{\rm g}$ = 0 V at different temperatures in linear (left) and log (right) scales. The experimental points are fitted with the Eq. (S1) from Ref. 14. The inset shows the fit parameters. **b** Log-log plot of $I_{\rm d}^{\rm avg}/T^{\alpha+1}$ versus $eV_{\rm d}/k_{\rm B}T$. **c** The same plot as in **a** but with the fit at fixed value of parameter $I_0 = 10^{-20}$ A/K$^{\alpha+1}$m^{-1}.

is about half that in the dense network of 5-AGNRs. [14] With the larger values of I_0, the fitting of our data becomes poor, as shown in Supplementary Figure 10c, where the parameter I_0 was fixed at 10^{-20} A/K$^{\alpha+1}$m^{-1}. Therefore, we believe that this difference of about eighteen orders of magnitude indicates a conduction mechanism other than hopping induced by nuclear tunneling of polarons.

Supplementary Note 7. Transport characterization of Li doped GNR heterojunctions

Each Li dose evaporated during the transport experiments corresponds to a tenth of the Li dose used in the ARPES experiment. The doping induced by a single Li dose during transport experiments is thus expected to be 0.017 electrons per 14-AGNR unit cell. This value can be converted to the position of E_{F} relative to the $\mathrm{CB}_1^{14-\mathrm{AGNR}}$ edge of 4 meV (estimated by the model for pristine sample) and 21, 45 and 74 meV after 1 – 3 Li dosages, respectively. The non-linear scaling of E_{F} with each Li dose is a result of the variation in density of states near the van Hove singularity at the $\mathrm{CB}_1^{14-\mathrm{AGNR}}$ band edge. These Fermi level shifts are in good agreement with those inferred from our transport model. The slightly lower values inferred from ARPES data might be due to the partial charge transfer to the metallic Au(788) substrate.

Supplementary Figure 11 presents the transport measurements of the $L = 500$ nm device during Li doping. We used identical Li doses as for the $L = 200$ nm device in the main text. Both devices demonstrate a similar behaviour – strong modulation of the drain current by Li doping and the suppression of modulation of hole conduction. A comparison of the experimental I_{d}-V_{d} curves to the fit using our tunneling model yields good agreement (Supplementary Figure 11a). According to the fit, for the $L = 500$ nm device the E_{F} shifts from 2 meV for the pristine sample to 120 meV after the third Li dose, which is in good agreement to the fit for the $L = 200$ nm device, presented in the main text. The bottom panels in Supplementary Figures 11a,b show the gate leakage current I_{g} measured simultaneously. The leakage current was monitored in all measurements and its value was negligible compared to I_{d} and it is not affected by Li doping.

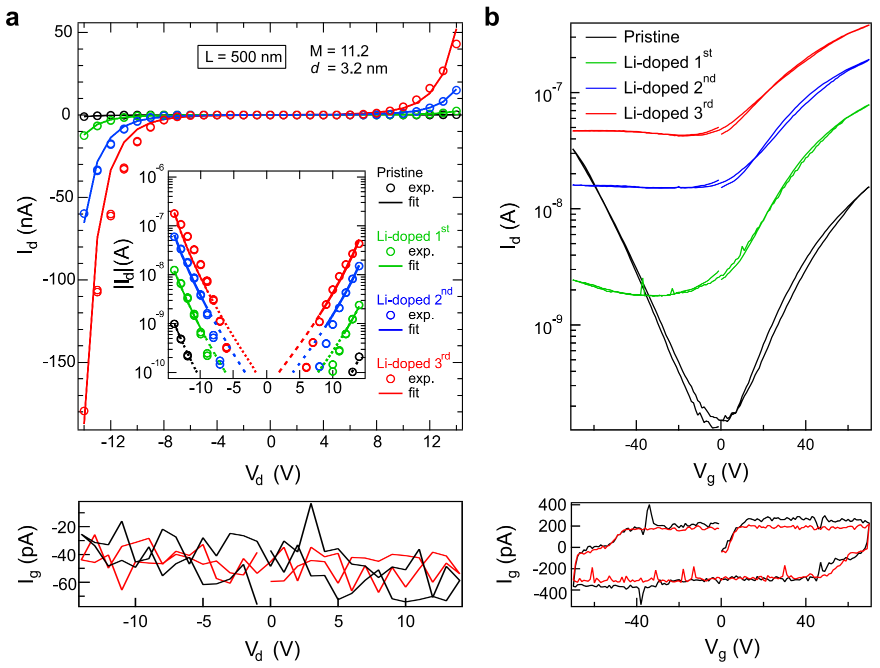

Supplementary Figure 11 Transport characteristics of the L = 500 nm device upon Li doping. a I_d-V_d curves at V_g = 0 in linear and log (insert) scales before (pristine) and after deposition of three identical Li doses ($\sim$ 0.1 Å each). Experimental points (exp.) are shown by circles, the fit is indicated by solid lines. **b** I_d-V_g characteristics at V_d = 14 V for three Li doses. The bottom panels in **a** and **b** shows the gate leakage current I_g of the device before (black) and after (red) deposition of three Li doses. The temperature of the sample is 4 K.

Supplementary Note 8. Li doping of the graphene device

To confirm that our Li doping of GNR heterojunction FETs was performed correctly, we conducted similar experiments on Li doping of large area graphene. Graphene was grown by chemical vapour deposition (CVD) method on an Ir(111) crystal and subsequently transferred onto 300 nm SiO_2/Si by electrochemical delamination. [16] We performed four-point transport measurements of the graphene device in our UHV system [16] upon deposition of identical Li doses as for GNR heterojunction FETs. The resulting I-V_g curves are shown in Supplementary Figure 12. After the first Li dose the gate potential required to reach the charge neutrality point (the point of minimum current) shifts towards negative voltages by $\sim$ 80 V. Such a large shift corresponds to the highest shift obtained by potassium doping graphene in a previous work. [17] Interestingly we can still observe bipolar behaviour after the first Li dose. Increasing the Li dosage, we do not observe current modulation for hole conduction. After deposition of the second and third Li doses, a plateau in the current emerges for $V_g < 80$ V. This observation is akin to what we have found for GNRs heterojunctions.

Supplementary References

1. Ruffieux, P. et al. Electronic Structure of Atomically Precise Graphene Nanoribbons. ACS Nano **6**, 6930–6935 (2012). URL `https://doi.org/10.1021/nn3021376`.

2. Ma, C. et al. Seamless Staircase Electrical Contact to Semiconducting Graphene Nanoribbons. Nano Lett. **17**, 6241–6247 (2017). URL

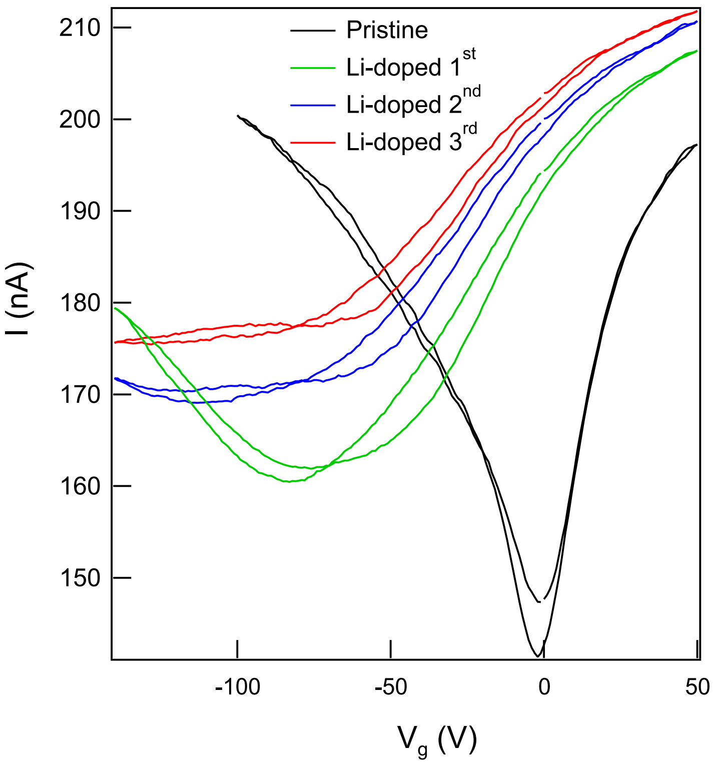

Supplementary Figure 12 Li doping of the graphene device. I-V_g characteristics of the graphene device upon Li doping at 4 K measured in four-point geometry (source voltage = 5 mV). The Li doses was the same as for the GNR heterojunction devices ($\sim$ 0.1 Å each).

https://doi.org/10.1021/acs.nanolett.7b02938.

3. Wang, S. et al. Quantum Dots in Graphene Nanoribbons. Nano Lett. **17**, 4277–4283 (2017). URL https://doi.org/10.1021/acs.nanolett.7b01244.

4. Senkovskiy, B. V. et al. Finding the Hidden Valence Band of N=7 Armchair Graphene Nanoribbons with Angle-Resolved Photoemission Spectroscopy. 2D Mater. **5**, 035007 (2018). URL https://doi.org/10.1088/2053-1583/aabb70.

5. Simonov, K. A. et al. Effect of electron injection in copper-contacted graphene nanoribbons. Nano Research **9**, 2735–2746 (2016). URL https://doi.org/10.1007/s12274-016-1162-2.

6. Huang, H. et al. Spatially Resolved Electronic Structures of Atomically Precise Armchair Graphene Nanoribbons. Sci. Rep. **2**, 983 (2012). URL https://doi.org/10.1038/srep00983.

7. Senkovskiy, B. V. et al. Boron-Doped Graphene Nanoribbons: Electronic Structure and Raman Fingerprint. ACS Nano **12**, 7571–7582 (2018). URL https://doi.org/10.1021/acsnano.8b04125.

8. Denk, R. et al. Exciton-dominated Optical Response of Ultra-narrow Graphene Nanoribbons. Nat. Commun. **5**, 4253 (2014). URL https://doi.org/10.1038/ncomms5253.

9. Senkovskiy, B. V. et al. Making Graphene Nanoribbons Photoluminescent. Nano Lett. **17**, 4029–4037 (2017). URL https://doi.org/10.1021/acs.nanolett.7b00147.

10. Overbeck, J. et al. A Universal Length-Dependent Vibrational Mode in Graphene Nanoribbons. ACS Nano **13**, 13083–13091 (2019). URL https://doi.org/10.1021/acsnano.9b05817.

11. Tries, A. et al. Hysteresis in graphene nanoribbon field-effect devices. Physical Chemistry Chemical Physics **22**, 5667–5672 (2020). URL http://dx.doi.org/10.1039/D0CP00298D.

12. Pollak, M. & Riess, I. A percolation treatment of high-field hopping transport. Journal of Physics C: Solid State Physics **9**, 2339–2352 (1976). URL https://iopscience.iop.org/article/10.1088/0022-3719/9/12/017.

13. Asadi, K. et al. Polaron hopping mediated by nuclear tunnelling in semiconducting polymers at high carrier density. Nature Communications **4**, 1710 (2013). URL https://doi.org/10.1038/ncomms2708.

14. Richter, N. et al. Charge transport mechanism in networks of armchair graphene nanoribbons. Sci. Rep. **10**, 1988 (2020). URL https://doi.org/10.1038/s41598-020-58660-w.

15. Kharche, N. & Meunier, V. Width and Crystal Orientation Dependent Band Gap Renormalization in Substrate-Supported Graphene Nanoribbons. J. Phys. Chem. Lett. **7**, 1526–1533 (2016). URL https://doi.org/10.1021/acs.jpclett.6b00422.

16. Hell, M. G. et al. Combined Ultra High Vacuum Raman and Electronic Transport Characterization of Large-Area Graphene on SiO_2. physica status solidi (b) **255**, 1800456 (2018). URL https://onlinelibrary.wiley.com/doi/abs/10.1002/pssb.201800456.

17. Chen, J.-H. et al. Charged-impurity scattering in graphene. Nat. Phys. **4**, 377–381 (2008). URL https://doi.org/10.1038/nphys935.
